# Genetic architecture of node-specific leaf length and candidate gene identification in maize revealed by a temperate–tropical multiparental population

**DOI:** 10.1186/s12870-026-09606-3

**Published:** 2026-08-04

**Authors:** Haoran Lyu, Fuyan Jiang, Yuxiang Luo, Ranjan K. Shaw, Guohong Wang, Xingming Fan

**Affiliations:** 1 College of Agriculture, Yunnan University, Kunming, 650500 China; 2 Institute of Food Crops, Yunnan Academy of Agricultural Sciences, Kunming, 650205 China

**Keywords:** Maize, Multi-parent population, Leaf length, GWAS, QTL mapping, Candidate genes

## Abstract

**Background:**

Optimizing leaf morphology is essential for improving maize plant architecture, plant density tolerance, and yield. Leaf length is a key agronomic trait controlled by complex genetic mechanisms. In this study, upper leaf length (ULL), ear leaf length (ELL) and lower leaf length (DLL) were evaluated across two environments using a multiparent RIL population derived from crosses between the temperate inbred line Ye107 and three tropical inbred lines. Combined with high-density GBS markers, genome-wide association study (GWAS) and QTL mapping were integrated to dissect the genetic basis of leaf length.

**Results:**

Leaf length traits exhibited high heritability (54.85%–76.85%). A total of 133 significant SNPs were identified by GWAS, and 13 candidate regions were detected by QTL localization, among which *ql1-17* explained 12.10% of the phenotypic variation. Joint analysis revealed multiple colocalized regions on chromosomes 1, 6 and 8, resulting in the identification of three key candidate genes *ZmHUA2 (Zm00001d029223)*,* ZmCAMTA5 (Zm00001d025235)*, and *ZmPAT16 (Zm00001d038367)*. Haplotype and qRT-PCR analyses showed that favorable haplotypes from tropical parents significantly increased leaf length, and these genes exhibited high expression specificity in the leaf elongation region.

**Conclusions:**

This study elucidates the complex genetic architecture of leaf length in temperate × tropical hybrid populations. Through integrated analyses, we identified key genomic loci and prioritized three candidate genes, particularly *ZmHUA2 (Zm00001d029223)* and *ZmCAMTA5 (Zm00001d025235)*. These findings provide valuable genomic resources and favorable alleles for marker-assisted selection, establishing a robust theoretical and practical foundation for maize ideotype breeding aimed at optimizing leaf morphology to enhance yield potential under high-density planting conditions.

**Supplementary Information:**

The online version contains supplementary material available at https://doi.org/10.1186/s12870-026-09606-3.

## Introduction

Maize (*Zea mays* L.) is one of the most important crops for food, feed, and industrial raw material in the world, and its stable yield improvement is crucial to ensure global food security [1]. With the continuous growth of the global population and the increasing scarcity of arable land resources, improving yield per unit area through increased planting density has become a core strategy of maize breeding [2, 3]. Over the past century, the remarkable increase in maize yield has largely resulted from a steady increase in planting density. The planting density in the U.S. maize belt has increased approximately from 30,000 plants ha⁻¹ in the 1930s to more than 80,000 plants ha⁻¹ at present [1]. Therefore, developing maize varieties with an ideal plant architecture (IPA) that adapts to dense planting has become a key objective of modern genetic improvement.

Plant architecture is a complex trait determined by multiple agronomic traits, such as plant height, ear position, leaf morphology and spatial distribution. As the primary organ for photosynthesis, leaf length, angle, and spatial arrangement directly influence canopy structure and light-use efficiency [4]. Appropriate leaf morphology can improve canopy light distribution and photosynthetic efficiency, thereby contributing to grain yield [4–7]. Therefore, leaf length is not only a key component of plant architecture in maize but also a key agronomic trait affecting photosynthetic efficiency and final yield.

Previous studies have identified numerous key regulators underlying maize ideal plant architecture (IPA). Classical liguleless genes, such as *lg1* and *lg2*, play central roles in leaf angle formation and canopy structure [8, 9]. In addition, *UPA1* and *UPA2* were reported to regulate upright plant architecture and contribute to high-density adaptation [10, 11]. Several dwarf-related genes affecting gibberellin biosynthesis or signaling have also been widely utilized to optimize plant height and lodging resistance [12, 13]. Moreover, members of the Growth-Regulating Factor (*GRF*) family participate in leaf growth and elongation by regulating cell proliferation and expansion [14, 15]. Other studies have further revealed that plant architecture is coordinately controlled by multiple developmental pathways involving hormone signaling, light response, and transcriptional regulation [16, 17]. Despite these advances, most previous studies mainly focused on leaf angle, plant height, or overall leaf morphology, whereas the genetic basis of node-specific leaf length remains insufficiently understood, particularly in temperate × tropical hybrid populations.

Maize leaf length is a typical quantitative trait, controlled by multiple genes and strongly influenced by G×E interaction [18–20]. Traditional genetic approaches, such as QTL mapping in biparental populations, have identified several genomic regions associated with leaf length. However, their mapping resolution and allelic diversity are often limited due to the narrow genetic background and low recombination frequency [21, 22]. With advances in high-throughput sequencing, genome-wide association study (GWAS) has become a powerful tool for elucidating the genetic basis of complex traits, enabling high-resolution detection of trait-associated loci across the genome [20, 23].

Despite these advances, several knowledge gaps remain. Most genetic analyses treat leaf length as a single overall trait, overlooking the developmental and functional differences among leaves at different nodes [24]. Whether the genetic control of upper, ear-position, and lower leaf length is shared or independent has not been systematically evaluated across environments [25]. In addition, temperate maize germplasm, such as the traditional Reid group, has undergone strong selection, resulting in serious genetic bottlenecks for traits related to dense planting adaption [26]. Recent studies have further confirmed the complexity and node specificity of the genetic architecture of maize leaf traits. Dai et al. (2023) reported both overlapped and independent regulatory loci for leaves at different nodes and identified a key candidate gene, *Zm00001d026491* [27]. Subsequently, Zeng et al. (2025) emphasized the importance of mining rare superior alleles to optimize ideal plant architecture using multi-parent population-based genomic prediction [28]. However, in temperate × tropical hybrid backgrounds, systematic multi-environment validation of the potential ‘decoupling’ of genetic effects among upper, ear-position, and lower leaf length using multiparent populations is still lacking.

To effectively integrate the high statistical power of linkage analysis with the high resolution of association analysis, multi-parent populations based on a “common parent × diversified donor” design have emerged as effective tools for dissecting complex quantitative traits [29]. Compared with traditional biparental populations, these populations capture richer allelic variation and genetic recombination [30]. Joint linkage-association analysis further improves the detection of small-effect loci and rare variants [31]. Previous studies have demonstrated that multi-parent populations enable more comprehensive identification of genetic loci controlling plant architecture traits such as leaf length and leaf posture [32, 33].

In this study, a “temperate × tropical” multi-parent population (MPP) containing three tropical-derived subpopulations was constructed. Using joint linkage and association analyses, we aimed to dissect the genetic architecture of the upper, ear-position, and lower leaf length in maize. The objectives of this study were to (1) identify genomic loci and QTL intervals significantly associated with leaf length; (2) detect key candidate genes regulating leaf length; (3) analyze the genetic effects of superior alleles. The results will enhance our understanding of the molecular mechanisms underlying maize plant architecture, and provide valuable genetic resources and theoretical support for improving leaf traits and developing maize varieties with ideal architecture for high-density planting through molecular design.

## Materials and methods

### Experimental materials and Population development

To analyze the genetic architecture of upper leaf length (ULL), ear leaf length (ELL), and lower leaf length (DLL) in maize, a multi-parent population (MPP) was constructed and used in this study. Three elite tropical inbred lines, YML46, CML444 and NK40-1, were crossed with a common male parent, Ye107, to generate three biparental populations with diverse genetic backgrounds. The F₁ plants from each cross were advanced by single seed descent (SSD) to the F₈ generation, resulting in the formation of an MPP containing three RIL subpopulations. The MPP consisted of 658 RILs, including Pop1 (YML46 × Ye107) with 216 RILs, Pop2 (CML444 × Ye107) with 221 RILs, and Pop3 (NK40-1 × Ye107) with 221 RILs (Table 1). The incorporation of diverse tropical germplasm generated rich genetic variation and wide phenotypic segregation, providing valuable materials for dissecting the genetic regulation of maize leaf length traits. For the GWAS analysis, the three RIL subpopulations were combined to form a diverse association panel consisting of 658 lines. This joint population structure allows for increased allelic diversity and mapping resolution by capturing recombination events across different tropical backgrounds. The genetic relationships and population stratification within this panel were accounted for using a kinship matrix (K) and principal components (PCA) as covariates in the mixed linear model.

**Table 1:** Details of parental lines used to develop the MPP population

Parent	Pedigree	Ecological type	ULL (cm)	ELL (cm)	DLL (cm)	Subpopulations	Number of RILs
Ye107	Derived from US hybrid DeKalb XL80	Temperate	44.87	48.98	49.78	-	-
YML46	Suwan1-1-1-2-1-2-1	Tropical	76.52	80.25	80.40	Pop1 (YML46 × Ye107)	216
CML444	P43C9-1-1-1-1-1-BBBB-1-1-2-5-1(DH)	Tropical	86.30	94.50	99.96	Pop2 (CML444 × Ye107)	221
NK40-1	Derived from US hybrid	Tropical	56.92	64.54	70.90	Pop3 (NK40-1 × Ye107)	221

### Field experiment design and phenotyping

To evaluate the environmental stability of genetic loci, Field experiments were conducted at two locations with similar ecological conditions. These locations included Dehong Dai Jingpo Autonomous Prefecture, Yunnan Province, (24.4°N, 98.6°E, approximately 920 m above sea level) in 2018 (designated as 18DH), and Baoshan City, Yunnan Province (24.9°N, 98.9°E, approximately 1650 m above sea level) in 2019 (designated as 19BS). All materials were planted in two-row plots, each consisting of two rows 4 m in length, with a row spacing of 0.7 m and a plant spacing of 0.25 m. Standard agronomic practices were followed throughout the maize growing season according to local cultivation guidelines.

Phenotypic data were collected approximately 20 days after silking, when vegetative growth was largely completed and leaf morphology had stabilized [34]. Five representative plants were randomly selected from each plot for measurement. The leaf length traits recorded included upper leaf length (ULL; the first leaf above the ear), ear leaf length (ELL; the leaf subtending the ear), and lower leaf length (DLL; the first leaf below the ear). Leaf length was measured using a meter ruler, and the phenotypic value of each plot was calculated as the mean of five plants and expressed in centimeters (cm).

## Statistical analysis of phenotypic data

To separate genetic effects from environmental influences and accurately evaluate the phenotypic performance of each RIL, a mixed linear model (MLM) was used to analyze the leaf length data. Best linear unbiased predictions (BLUPs) for each trait across individual and multiple environments were calculated using the lme4 package in R (R Core Team, 2025). The model was specified as:

$$y={X}\beta+{Z}\mu+\epsilon$$

where *y* represents the vector of observed phenotypic values; * X* represents the design matrix for fixed effects; $\:\beta$ is the vector of the fixed-effect parameters; * Z* represents the design matrix for random effects; *μ* is the vector of random effect parameters; and *ε* is the vector of residual error representing unexplained variation [35]. The cross-environmental BLUP values, which account for environmental variation, were used as genetic estimates after subsequent genome-wide association analysis and QTL mapping studies.

To evaluate the genetic stability of the traits, broad-sense heritability (H^2^) was estimated based on variance components obtained from a multi-environment analysis of variance (ANOVA). Heritability was calculated as:

$${H}^{2}=\frac{{\sigma}_{g}^{2}}{{\sigma}_{g}^{2}+\frac{{\sigma}_{ge}^{2}}{e}}\times100\%$$

where ${\sigma}_{g}^{2}$ represents the genotype variance; ${\sigma}_{ge}^{2}$ represents the genotype × environment interaction variance; and *e* represents the number of environments [36].

## DNA extraction, GBS library construction, sequencing, and SNP identification

During the maize seedling stage, fresh and healthy leaf tissue was collected from each RIL and parental lines for genomic DNA extraction. High-quality genomic DNA was extracted using the CTAB (Cetyltrimethylammonium Bromide) method [37]. DNA concentration and purity (A260/A280 ratio) were measured using a NanoDrop 2000 spectrophotometer, and DNA integrity was assessed by 1% agarose gel electrophoresis.

Qualified DNA samples were used for genotyping-by-sequencing (GBS) library construction. Genomic DNA was double-digested with the restriction enzymes PstI and MspI. Barcode adaptors were ligated to the digested fragments, followed by purification and PCR amplification to enrich adaptor-ligated fragments. The constructed GBS libraries were sequenced using the Illumina NovaSeq 6000 platform (Illumina, USA). A total of 1251.2 Gb of high-quality clean data was generated from the 662 samples (658 RILs and 4 parental lines). Each sample yielded an average of 1.89 Gb of clean data, corresponding to a mean genome-wide sequencing depth of 6.34×. Raw sequencing data were processed by removing adaptor sequences and low-quality reads. Clean reads were then aligned to the maize B73 reference genome (RefGen_v4) using BWA (Burrows-Wheeler Aligner) [38]. The alignment results were converted into BAM format using SAMtools, followed by sorting and removal of duplicated reads [39]. SNPs (Single Nucleotide Polymorphisms) were identified based on the aligned data. To ensure marker quality for downstream analyses, SNPs with a missing rate greater than 20% and a minor allele frequency (MAF) less than 5% were filtered out [40]. The final high-quality SNP set was annotated using ANNOVAR to determine their locations (e.g., intergenic regions, exons, introns, etc.) and potential functional effects (e.g., synonymous or non-synonymous variants) [41].

## Phylogenetic trees, principal component analysis (PCA), population structure and linkage disequilibrium analysis

To assess the genetic differentiation within the population and obtain covariates for correcting population stratification in GWAS, principal component analysis (PCA) was performed using GCTA (v1.5.0) [42]. A genetic relationship matrix was calculated based on SNP data, and the first few major principal components (PCs) were extracted. The PCA results were used to visualize the genetic clustering of RILs and were included as fixed-effect covariates in the mixed linear model (MLM).

Population structure and ancestral genetic composition was inferred using the model-based Bayesian clustering algorithm implemented in ADMIXTURE (v1.3.0) [43]. The number of ancestral populations (K) was set from 1 to 10, and the optimal K value was determined based on cross-validation error.

To further characterize the genetic relationship among RILs, a phylogenetic tree was constructed. Pairwise genetic distances were calculated using TreeBeST (v1.9.2), and an unrooted phylogenetic tree was constructed using the neighbor-joining (NJ) method. The reliability of tree branches was assessed with 1,000 bootstrap iterations [44]. Linkage disequilibrium (LD) levels and genome-wide LD decay patterns were analyzed using PopLDdecay (v3.42) [45]. The squared correlation coefficient (r^2^) was calculated for all SNP pairs across the ten maize chromosomes, and the average r^2^ was plotted against physical distance (kb) to estimate LD decay. The physical distance at which LD decayed to a defined threshold was used as a reference for interpreting association signals and determining candidate gene search intervals in subsequent GWAS.

### Genome-wide association analysis (GWAS)

To identify genomic regions significantly associated with maize leaf length traits, genome-wide association analysis was performed using GEMMA (Genome-wide Efficient Mixed Model Association). The analysis was conducted using high-quality SNP markers and phenotypic BLUP values obtained across environments [46]. A mixed linear model (MLM) was applied to effectively control false positive associations caused by population structure and relatedness.

Population structure was accounted for using principal component analysis. The first three principal components (PC1, PC2, and PC3) were included as fixed-effect covariates in the MLM. The kinship matrix (K-matrix) was calculated in GEMMA using all high-quality SNP markers and incorporated into the model as a random effect to account for genetic relatedness among individuals.

The genome-wide significance threshold was determined by balancing statistical stringency and detection power. Although Bonferroni, false discovery rate (FDR), or effective marker number (*M*_*eff*_)-based corrections provide strict control of Type I error, these methods generally assume partial or complete independence among tests [47, 48]. In plant genomes with extensive linkage disequilibrium (LD), SNP markers are often correlated, resulting in an overestimation of the effective number of independent tests and consequently an overly conservative threshold [49]. Furthermore, complex quantitative traits are typically controlled by numerous loci with small effects, and excessively stringent significance thresholds may substantially reduce statistical power and increase Type II error [50, 51]. Therefore, an empirical threshold of − log₁₀(p) > 4.5 was adopted in this study. Similar relaxed thresholds (− log₁₀(p) ≈ 4–5) have been widely applied in plant GWAS analyses of complex traits [52–54]. Importantly, GWAS signals were further filtered through colocalization with QTL identified from an independent linkage population, forming a dual-validation framework that enhances detection power while effectively controlling false positives. This strategy improves the reliability of association signals while minimizing false-positive associations.

The GWAS results were visualized using the CMplot package in R (v4.5.1) [55] which was used to generate Manhattan and Q-Q plots. The Manhattan plot displayed the genome-wide distribution of association signals, while the Q-Q plot evaluates model performance and detects potential systematic bias in the association results.

## Genetic linkage map construction and QTL mapping

To detect QTL in the three RIL subpopulations, high-density genetic linkage maps were constructed for each subpopulation. Polymorphic SNP markers segregating within each subpopulation were used to construct linkage groups using JoinMap (v4.0) [56]. Marker order within each linkage group was determined using the maximum likelihood algorithm, and recombination frequencies were converted into genetic distance (cM) using the Kosambi mapping function.

Based on the three genetic maps and the BLUP values of leaf length traits, QTL analysis was performed using Windows QTL Cartographer (v2.5). Composite interval mapping (CIM) was used to identify QTL [57]. The logarithm of odds (LOD) threshold for QTL detection was determined by 1000 permutation tests at a significance level of *P* < 0.05 [58]. For each detected QTL, the percentage of phenotypic variation explained (PVE) was estimated using the square of the partial correlation coefficient (R^2^) [57]. QTL were named according to the trait abbreviation, chromosome number, and detection order on each chromosome. For example, “*ql1-17*” represents the 17th leaf-length QTL identified on chromosome 1.

The multi-environmental experiment (MET) module in QTL IciMapping (v4.2) was used to analyze QTL × environment interactions. The analysis included the inclusive composite interval mapping method (ICIM-MET) to estimate the additive effect (A) and the additive × environment interaction effect (A×E). For each detected QTL, the contribution rates of the additive effect [PVE(A)] and the interaction effect [PVE(A×E)] were calculated. Following the ICIM-MET framework and previous QTL × environmental interaction studies, the relative contribution of the interaction effect was quantified as PVE(A×E)/PVE(A) [59, 60]. In this study, QTL were classified into three stable categories based on this ratio: < 10% as highly stable, 10–30% as stable, and > 40% as environmentally sensitive.

## Mining and functional prediction of candidate genes

Based on the results of LD decay analysis, the initial candidate gene search region was defined as 20 kb upstream and downstream of significant SNPs identified by GWAS. To improve the accuracy of candidate gene identification, priority was given to genomic regions where significant GWAS signals overlapped with QTL confidence intervals.

Gene annotation information within the target intervals was retrieved from the MaizeGDB and the NCBI Gene databases [61, 62]. For genes with incomplete functional annotation or annotated as proteins of unknown function, functional inference was performed using comparative genomics homology-based analysis [63]. Based on gene annotation and homology information, attention was given to genes involved in biological processes related to leaf development, including cell cycle regulation, cell wall synthesis, and plant hormone signaling pathways (such as auxin and gibberellin). According to these criteria, key candidate genes were selected for subsequent haplotype analysis and expression validation.

### Haplotype analysis of candidate genes

To further investigate the allele variation in key candidate genes identified through GWAS and QTL mapping and their effects on leaf length traits, haplotype analysis was performed. Haploview (v4.2) was used to construct haplotype blocks based on SNPs located within the coding regions (CDS) of candidate genes and their flanking regulatory regions [64].

The MPP population was grouped according to the haplotype of each RIL at the target gene. Phenotypic differences among haplotype groups were evaluated using BLUP values for leaf length traits. Statistical analyses were performed in R. Student’s t-test was used for comparison between two haplotypes, while one-way ANOVA followed by multiple comparison tests was applied when more than two haplotypes were present. Box plots were generated to visualize the phenotypic distributions associated with major haplotypes. The analysis aimed to identify superior haplotypes significantly associated with leaf length traits and to assess whether their effects were positive (increasing) or negative (decreasing).

### Relative expression analysis of candidate genes

To investigate the expression patterns of key candidate genes during leaf development and their potential regulatory roles in leaf length, spatiotemporal sampling was performed on leaf tissues at different nodal positions. qRT-PCR analyses were conducted using the parental maize inbred lines of the multiparent population, which exhibited contrasting leaf-length phenotypes. Each parental line was planted in 10 independent rows (plots), with seven plants per plot. Five representative plants were randomly selected from each plot for phenotypic observation and tissue collection. For gene expression analysis, three independent biological replicates were collected for each sampling point, and each replicate consisted of independently collected leaf tissues used for RNA extraction and qRT-PCR analysis. As the colocalization analysis primarily focused on upper and lower leaf length traits, sampling was conducted during critical developmental stages of these two leaf positions.

For the lower leaves, the fourth leaf (Leaf 4) was selected as a representative and sampled during steady-state elongation phase (approximately 3 days after the leaf tip emerged from the sheath, when the elongation rate becomes stable). Tissue samples were collected from two regions: 0.5 cm from the leaf base (representing the cell division zone) and 2–3 cm region above the base (representing the cell elongation zone) [65, 66]. For the upper leaves, the first leaf above the ear was selected. Sampling was conducted at the corresponding steady-state elongation stage to ensure developmental compatibility with the lower leaf. The same sampling regions (0.5 cm at the base and 2–3 cm above the base) were collected to maintain consistency between leaf positions [65, 67]. At least three biological replicates were collected at each sampling point [68]. All samples were immediately frozen in liquid nitrogen after collection and stored at -80 °C until RNA extraction.

The total RNA was extracted from each sample using the RNAprep Pure Plant Kit (Tiangen, Beijing, China). RNA concentration and purity were measured using a micro-spectrophotometer, and RNA integrity was verified by agarose gel electrophoresis. First strand cDNA was synthesized from total RNA using the FastKing RT Kit (Tiangen, Beijing, China), with genomic DNA contamination removed during the reaction. Gene-specific primers were designed using the NCBI Primer-BLAST tool (primer sequences are listed in Table S1). qRT-PCR was performed using the Archimed R4 real-time PCR system (AsnBio, Hangzhou, China) with the SuperReal PreMix Plus (SYBR Green) Kit (Tiangen, Beijing, China). The PCR conditions consisted of an initial denaturation at 95 °C for 15 min, followed by 40 cycles of denaturation at 95 °C for 10 s, annealing at 58 °C for 20 s, and extension at 72 °C for 20 s. Maize LUG and MEP genes were used as internal reference genes for normalization. Three technical replicates were performed for each biological replicate. The relative expression levels of genes were calculated using the 2^−ΔΔCt^ method [69].

## Results

### Phenotypic variation and heritability of leaf length traits

**Fig. 1:**
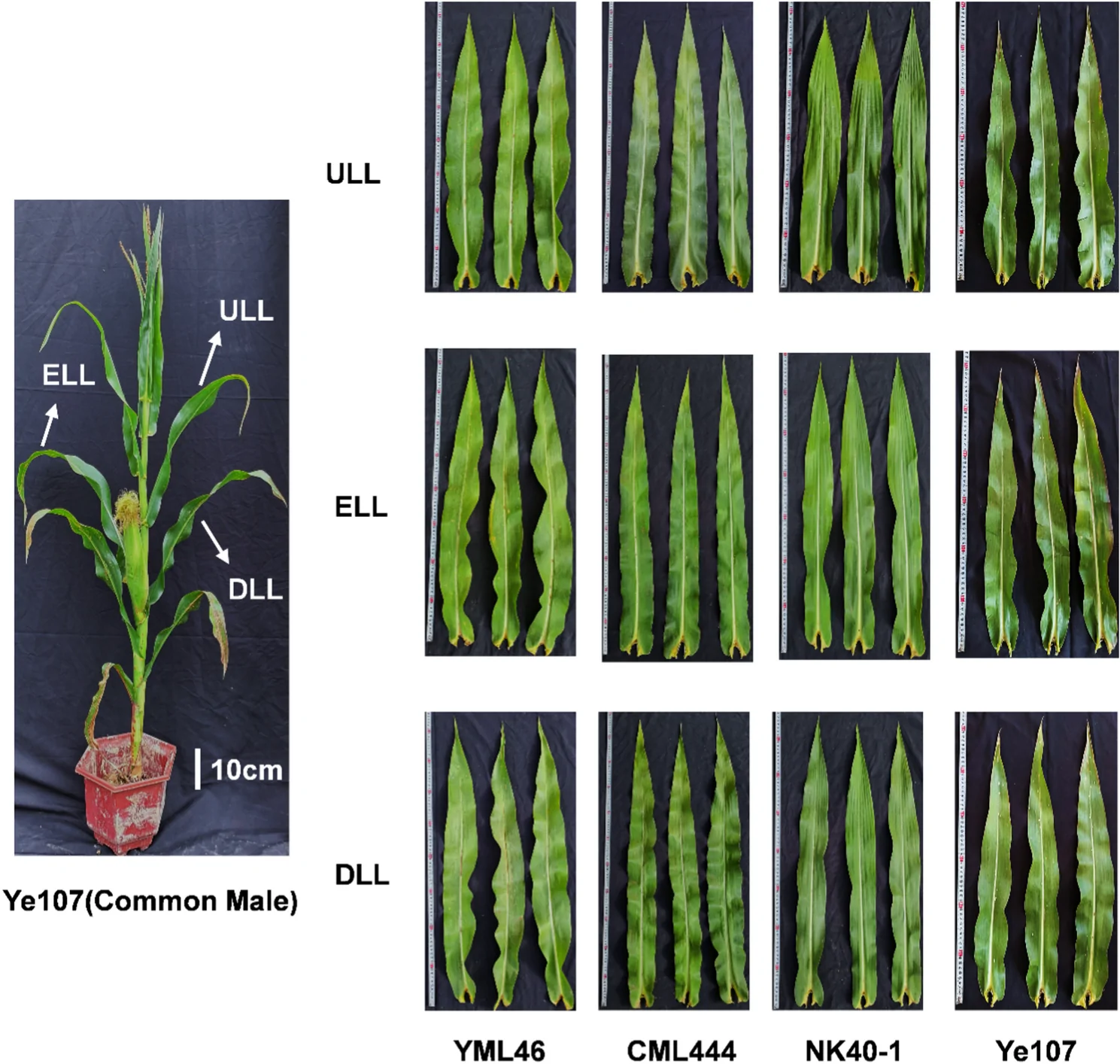
Morphological variation of leaf length traits in the four parental inbred lines. The morphology and sampling positions of leaves in the common parent Ye107 were shown, along with the comparative leaf morphology at different positions among Ye107, YML46, CML444, and NK40-1

**Fig. 2:**
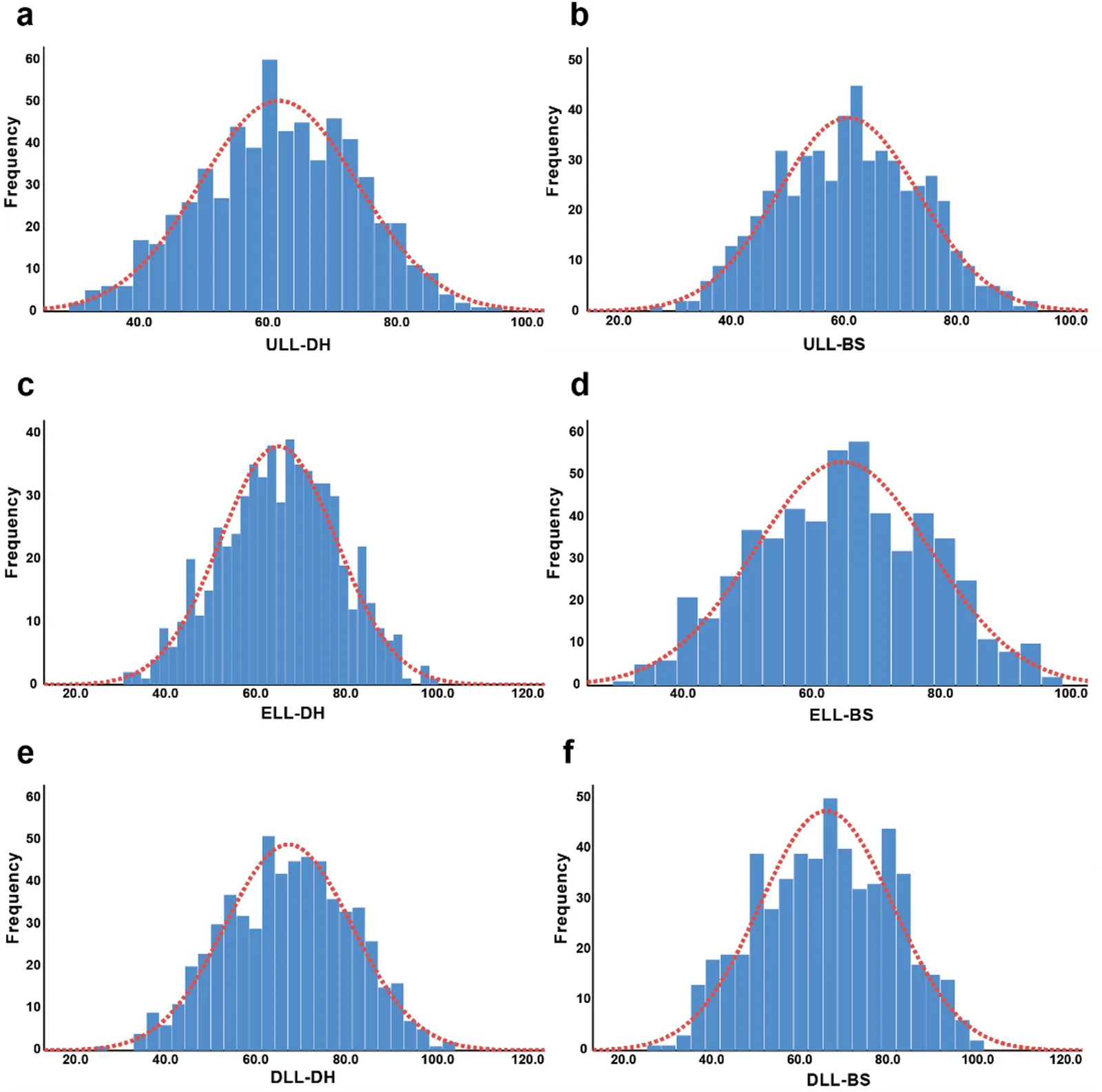
Frequency distribution of leaf length traits across environments (**a**) Upper leaf length (ULL) in the 18DH environment; (**b**) Upper leaf length (ULL) in 19BS environment; (**c**) Ear leaf length (ELL) in the 18DH environment; (**d**) Ear leaf length (ELL) in the 19BS environment; (**e**) Lower leaf length (DLL) in the 18DH environment; (**f**) Lower leaf length (DLL) in the 19BS environment

The upper leaf length (ULL), ear leaf length (ELL), and lower leaf length (DLL) exhibited wide and stable phenotypic variation across the three subpopulations under both environments (18DH and 19BS) (Table 2). As shown in Fig. 1, the common parent Ye107 and the three female parents (YML46, CML444 and NK40-1) showed clear morphological differences at the three leaf positions, providing a genetic basis for the extensive variation in the MPP. The coefficient of variation (CV) for all traits ranged from 0.14 to 0.20, indicating substantial phenotypic variability suitable for genetic analysis. The distribution analysis showed skewness values ranging from − 0.672 to 0.470 and Kurtosis values ranging from − 0.445 to 1.218, suggesting that the phenotypic data approximated a normal distribution across subpopulations and environments (Fig. 2). These results were consistent with the characteristics of complex quantitative traits.

All leaf length traits exhibited moderate to high broad-sense heritability (H^2^), ranging from 54.85% (DLL in Pop1) to 76.85% (ULL in Pop2). The relatively high heritability values (H^2^> 50%) indicate that phenotypic variation was primarily controlled by genetic factors, with a comparatively smaller environmental influence. Consistent with this observation, significant positive correlation was detected between the two environments (18DH and 19BS) for all traits (Table 2). Although environmental conditions affected leaf growth to some extent, the combination of high heritability and strong cross-environment correlations indicate good environmental stability of leaf length across RIL families. This stability provides a reliable basis for subsequent GWAS and QTL analyses.

**Table 2:** Descriptive statistics of leaf length (LL) for the three subpopulations

Trait	Populations	Environment	Mean	Standard Deviation	Skewness	Kurtosis	Coefficient of Variation (CV)	H2 (%)	Correlation Coefficient (*r*)
ULL	Pop1	18DH	67.768	9.529	-0.16	0.268	0.14	65.80	0.498
		19BS	64.988	10.45	0.129	-0.117	0.16		
	Pop2	18DH	67.002	11.835	-0.445	0.066	0.18	76.85	0.618
		19BS	69.345	10.29	-0.268	0.027	0.15		
	Pop3	18DH	53.109	9.237	0.177	0.193	0.17	60.97	0.469
		19BS	52.291	9.373	0.225	-0.311	0.18		
ELL	Pop1	18DH	71.884	9.934	-0.128	0.64	0.14	63.61	0.452
		19BS	68.514	11.373	0.006	-0.153	0.17		
	Pop2	18DH	71.105	12.18	-0.230	-0.150	0.17	75.10	0.602
		19BS	74.698	10.59	-0.185	-0.006	0.14		
	Pop3	18DH	56.296	9.951	0.158	0.147	0.18	64.43	0.476
		19BS	55.020	10.002	0.262	-0.056	0.18		
DLL	Pop1	18DH	74.388	10.901	-0.008	-0.297	0.15	54.85	0.390
		19BS	69.630	12.502	-0.168	-0.381	0.18		
	Pop2	18DH	74.124	13.073	-0.338	-0.206	0.18	72.67	0.565
		19BS	77.592	11.744	-0.672	1.218	0.15		
	Pop3	18DH	57.477	10.936	0.262	0.359	0.19	67.32	0.511
		19BS	55.618	11.104	0.470	0.214	0.20		

### Identification, quality, and distribution of SNP markers

GBS sequencing data analysis of the MPP identified a total of 213,087 high-quality SNP markers after stringent quality filtering for subsequent analyses.

**Fig. 3:**
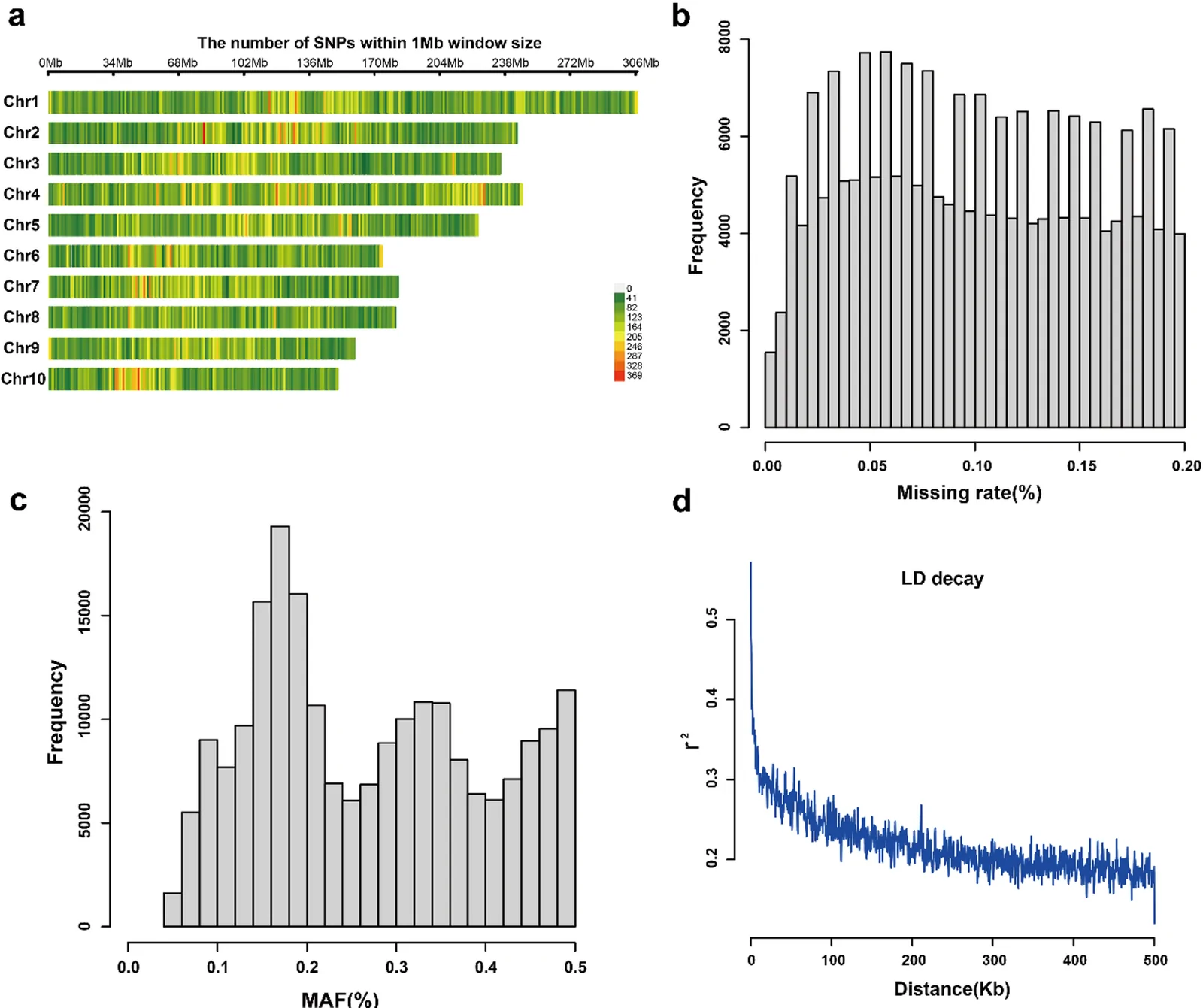
Genome-wide distribution and quality assessment of SNPs: (**a**) SNP density per 1 Mb window. (**b**) Distribution of SNP missing rates. (**c**) Minor allele frequency distribution; and (**d**) LD decay plot

The quality assessment of these SNPs is shown in Fig. 3. The distribution of missing rates indicated that the majority of SNPs had missing rates well below the 20% threshold, with most markers showing missing rates of less than 5% (Fig. 3b), indicating a high overall genotyping quality. The minor allele frequency (MAF) distribution showed that most SNPs were distributed within the range of 0.05 to 0.5, with a peak around 0.15–0.20 (Fig. 3c). These results indicate abundant genetic polymorphism in the MPP, providing a basis for subsequent association analyses.

Genome-wide linkage disequilibrium (LD) decay was evaluated using all high-quality SNPs (Fig. 3d). LD decreased rapidly with increasing physical distance. The mean r^2^ value exceeded 0.5 at short distances (< 10 kb) and decayed to approximately 0.2 at distances of about 100–150 kb, after which the decay became plateaued. The LD decay distance corresponding to r^2^ ≈ 0.32 was approximately 20 kb. Therefore, based on the genome-wide LD decay pattern observed in this multiparent population, a ± 20 kb range around significant SNPs was used for candidate gene identification. The relatively rapid LD decay observed in this population indicates a high level of historical recombination, which is advantageous for high-resolution genetic mapping and precise candidate gene screening.

### Population structure and genetic differentiation analysis

**Fig. 4:**
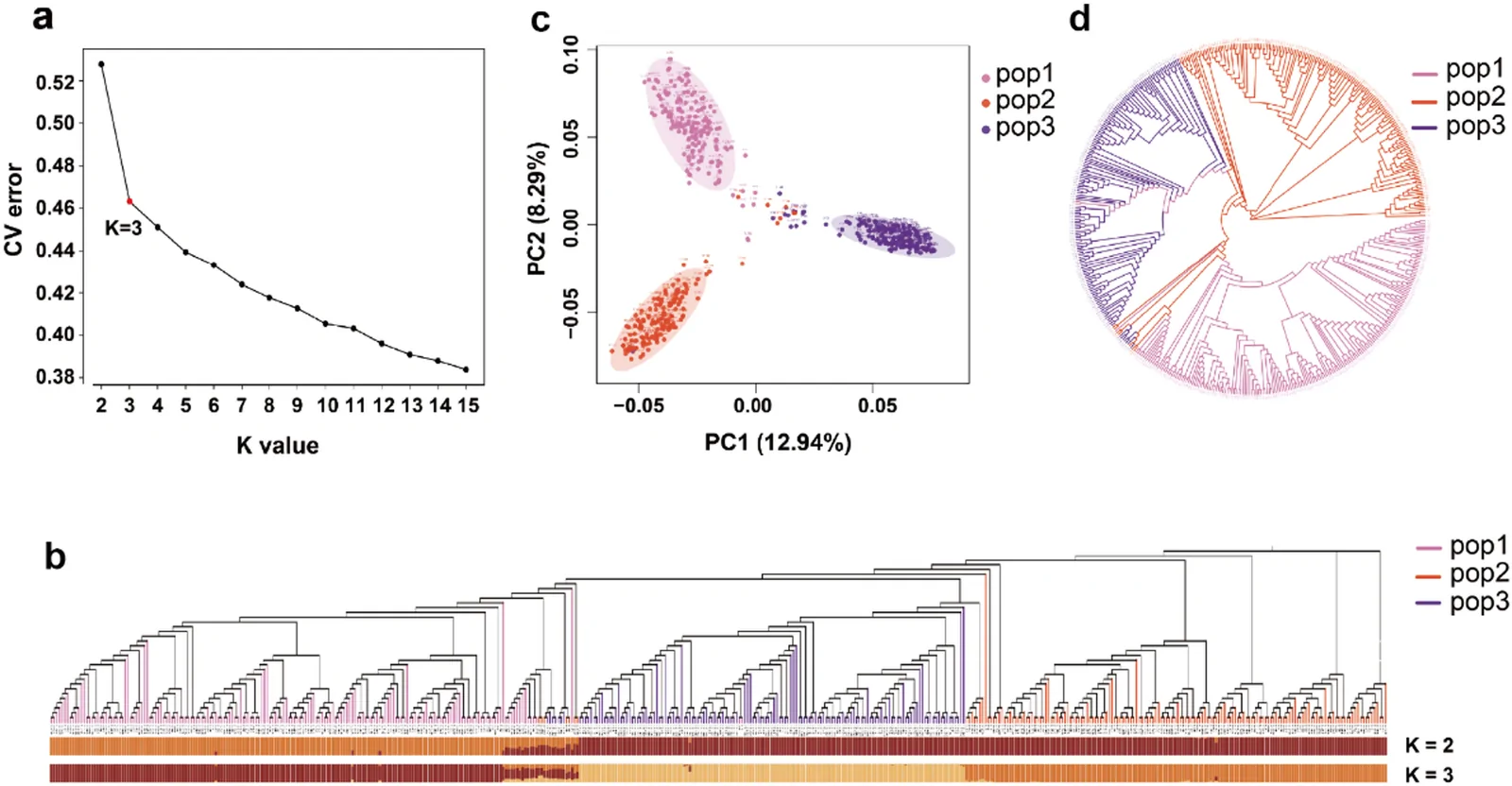
Population structure analysis of the MPP. (**a**) ADMIXTURE cross-validation error. (**b**) Bayesian cluster analysis at K = 3. (**c**) Principal component analysis. (**d**) Neighbor-joining phylogenetic tree

To evaluate the genetic background and population differentiation of the MPP, principal component analysis (PCA), phylogenetic analysis, and model-based clustering were performed using the high-quality SNP dataset (Fig. 4).

The population structure was inferred using ADMIXTURE. Cross-validation analysis indicated a clear inflection point in the CV error at K = 3, after which the rate of decrease became minimal. Therefore K = 3 was considered the optimal number of subgroups (Fig. 4a). The ancestry proportion plot at K = 3 (Fig. 4b) showed that almost all individuals exhibited predominant ancestry from a single group and were clearly divided into three clusters. These clusters corresponded to three RIL subpopulations: Pop1, Pop2, and Pop3, derived from the donor parents YML46, CML444, NK40-1, respectively. The PCA results supported this classification. Scatter plots based on the first two principal components (PC1 and PC2) showed that all 658 RILs were clearly divided into three distinct clusters (Fig. 4c). PC1 and PC2 explained 12.94% and 8.29% of the genetic variation, respectively. The clustering pattern was consistent with the three subpopulations, Pop1, Pop2, and Pop3. Similarly, the phylogenetic tree revealed three major clades corresponding to the three subpopulations (Fig. 4d), indicating distinct genetic background of each group.

Overall, the results of population structure, PCA, and phylogenetic analysis were highly consistent and demonstrated clear population stratification within the MPP. This well-defined structure should be accounted for subsequent genome-wide association analyses to avoid spurious associations.

### Identification of leaf length-associated SNPs through GWAS

**Fig. 5:**
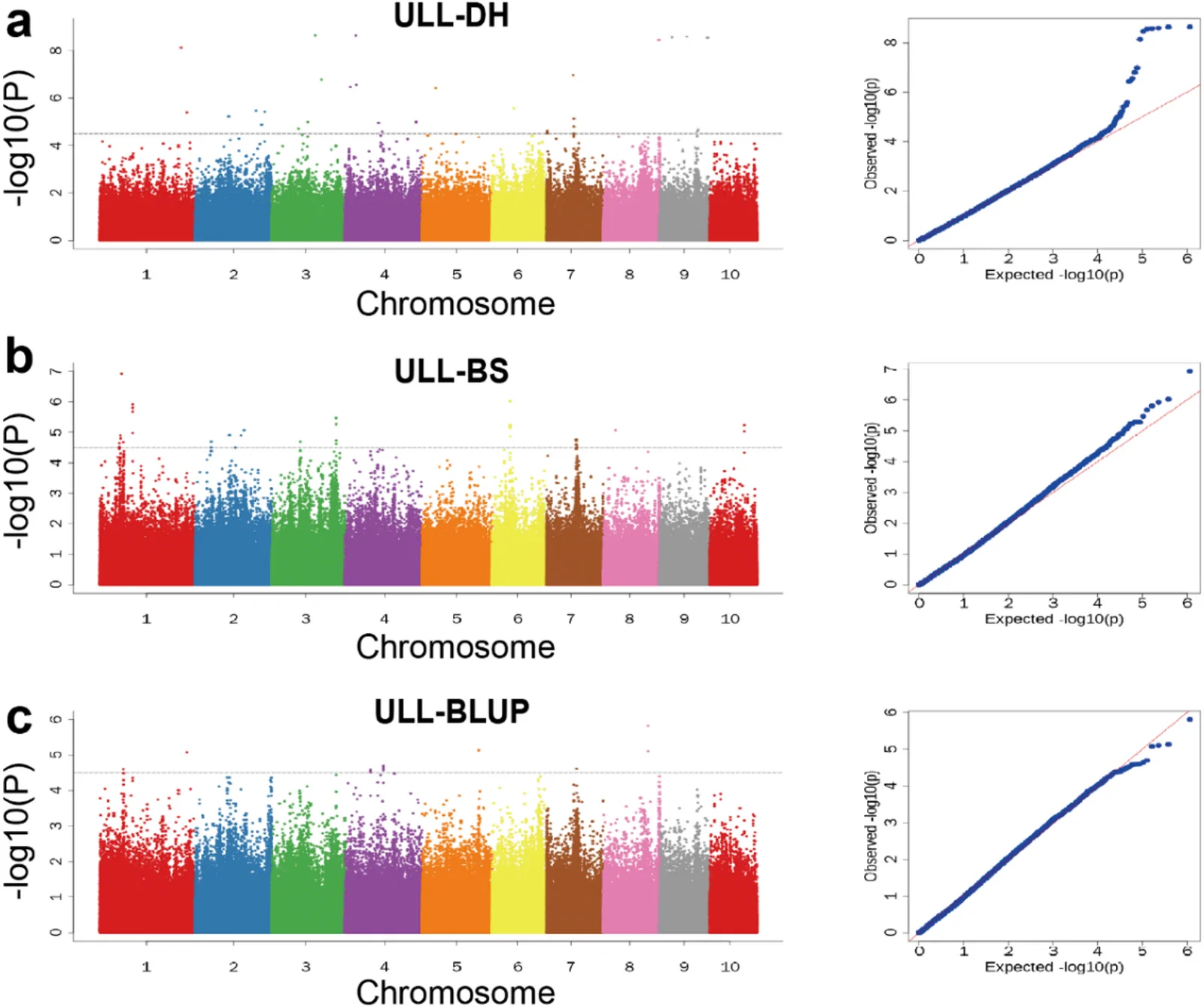
GWAS of upper leaf length. Manhattan plots (left) and Q-Q plots (right) of (**a**) the 18DH environment, (**b**) the 19BS environment, and (**c**) BLUP values

**Fig. 6:**
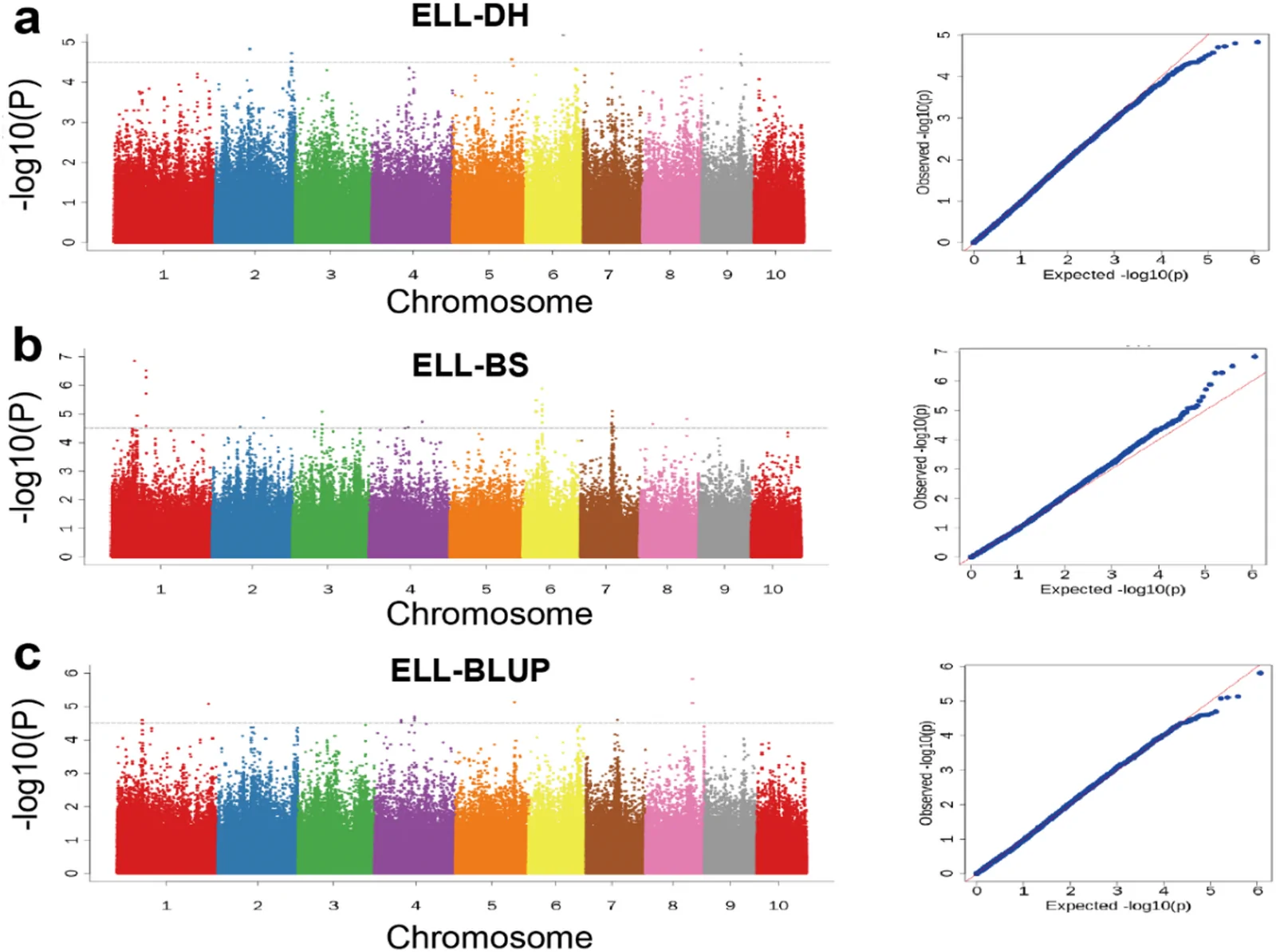
GWAS for ear leaf length. Manhattan plots (left) and Q-Q plots (right) for (**a**) the 8DH environment, (**b**) the 19BS environment, and (**c**) BLUP values

**Fig. 7:**
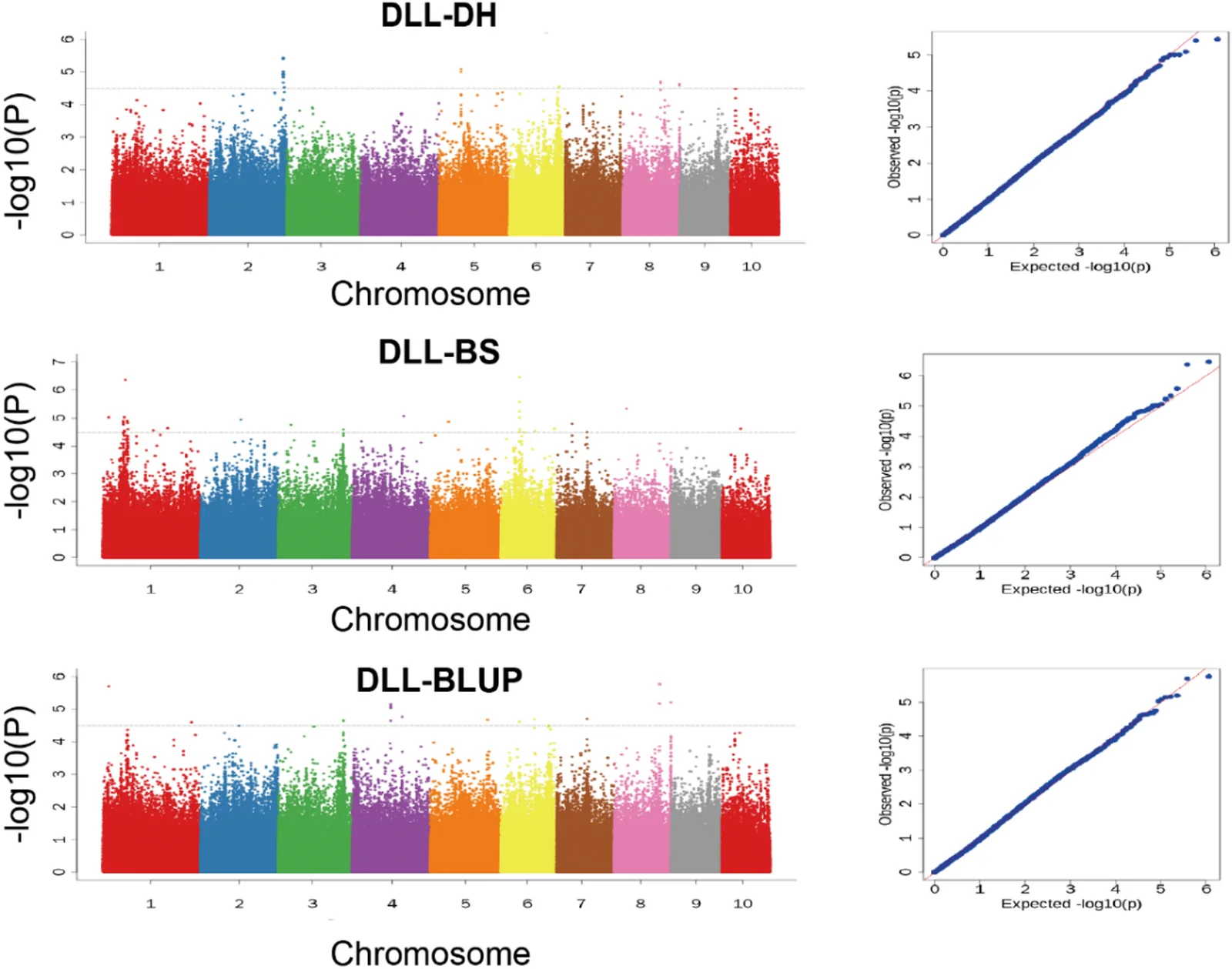
GWAS of lower leaf length. Manhattan plots (left) and Q-Q plots (right) for (**a**) the 18DH environment, (**b**) the 19BS environment, and (**c**) BLUP values

To identify genetic loci associated with leaf length traits, GWAS was performed using BLUP values for upper leaf length, ear leaf length and lower leaf length, as well as phenotypic data from the two environments (18DH and 19BS). The Q-Q plots showed that the observed -log10(p) values closely followed the expected distribution, with deviations observed only at the extreme (Figs. 5, 6 and 7). This pattern indicates that the mixed linear model effectively controlled for population structure and kinship, thereby reducing false-positive association. Using the genome-wide significance threshold, a total of 133 SNP loci significantly associated with leaf length traits were identified (Table S2). Manhattan plots illustrate the distribution of these signals across the ten maize chromosomes (Figs. 5, 6 and 7). Key SNPs that were consistently across environments and BLUP values are summarized in Table 3.

For upper leaf length, a stable association locus, SNP1-279508495 was detected on chromosome 1 (Fig. 5). This SNP showed significance in both the 18DH environment and the BLUP analysis (- log10(p) = 5.40), explaining 1.21% of the phenotypic variation, with an additive effect of 1.71. For ear leaf length, two key loci were identified (Fig. 6). SNP8-143674668 on chromosome 8 showed stability across environments and was detected in both the 19BS environment and BLUP analysis, with the strongest association signal (- log10(p) = 5.81) and an additive effect of 2.47. In addition, SNP5-180255939 on chromosome 5, detected in both the 18DH environment and the BLUP analysis, exhibited the largest negative additive effect (-4.55). For lower leaf length, six key SNPs were identified (Fig. 7). Among them, three loci showed cross-environmental stability and detected in both the 19BS environment, and BLUP analysis, with the significant p-value (- log10(p) = 5.69), and explaining 0.84% of the phenotypic variation. SNP3-206954273 on chromosome 3 showed a large positive additive effect (3.10), while SNP8-179883797 on chromosome 8 showed higher significance (- log10(p) = 5.20) in both the 18DH environment and BLUP analysis. Notably, SNP1-279508495 was associated with both upper and lower leaf length, suggesting that this genomic region may exhibit pleiotropic effects on leaf length at different plant positions or may contain tightly linked functional genes. Candidate gene screening within a 20 kb region upstream and downstream of the significant SNPs was conducted using Maize GDB, InterProt, UniProt, and NCBI databases. Three candidate genes, *ZmHUA2 (Zm00001d029223)*,* ZmCAMTA5 (Zm00001d025235)*, and *ZmPAT16 (Zm00001d038367)*, were identified as potential regulators of maize leaf length on chromosomes 1, 6, and 10, respectively (Table S3).

**Table 3:** SNPs significantly associated with leaf length (LL)

Trait	Environments	SNP	−log10 (*p*)	PVE	Add. effect
ULL	18DH, BLUP	1-279508495	5.40	1.21	1.71
	19BS	1-63031255	4.64	0.1	1.70
ELL	19BS, BLUP	8- 143,674,668	5.81	0.21	2.47
	18DH, BLUP	5- 180,255,939	5.13	0.28	-4.55
DLL	19BS, BLUP	1- 17,369,125	5.69	0.84	1.72
	19BS, BLUP	3- 206,954,273	4.64	0.69	3.10
	19BS	1- 63,031,328	4.89	0.33	1.59
	BLUP	1-279508495	4.60	0.24	-0.82
	18DH, BLUP	8-179883797	5.20	0.49	2.22

### QTL mapping

Using composite interval mapping (CIM), a total of 448 QTL tightly linked to maize leaf length were identified across different subpopulations and environments of the MPP (Table S4). Thirteen QTL were retained based on their co-localization with GWAS results (Table 4). These QTL were distributed on chromosomes 1, 6, 8, and 10 (Fig. S1). The LOD values ranged from 2.51 to 4.57, and individual QTL explained 1.03% to 12.82% of the phenotypic variation (Table 4).

**Fig. 8:**
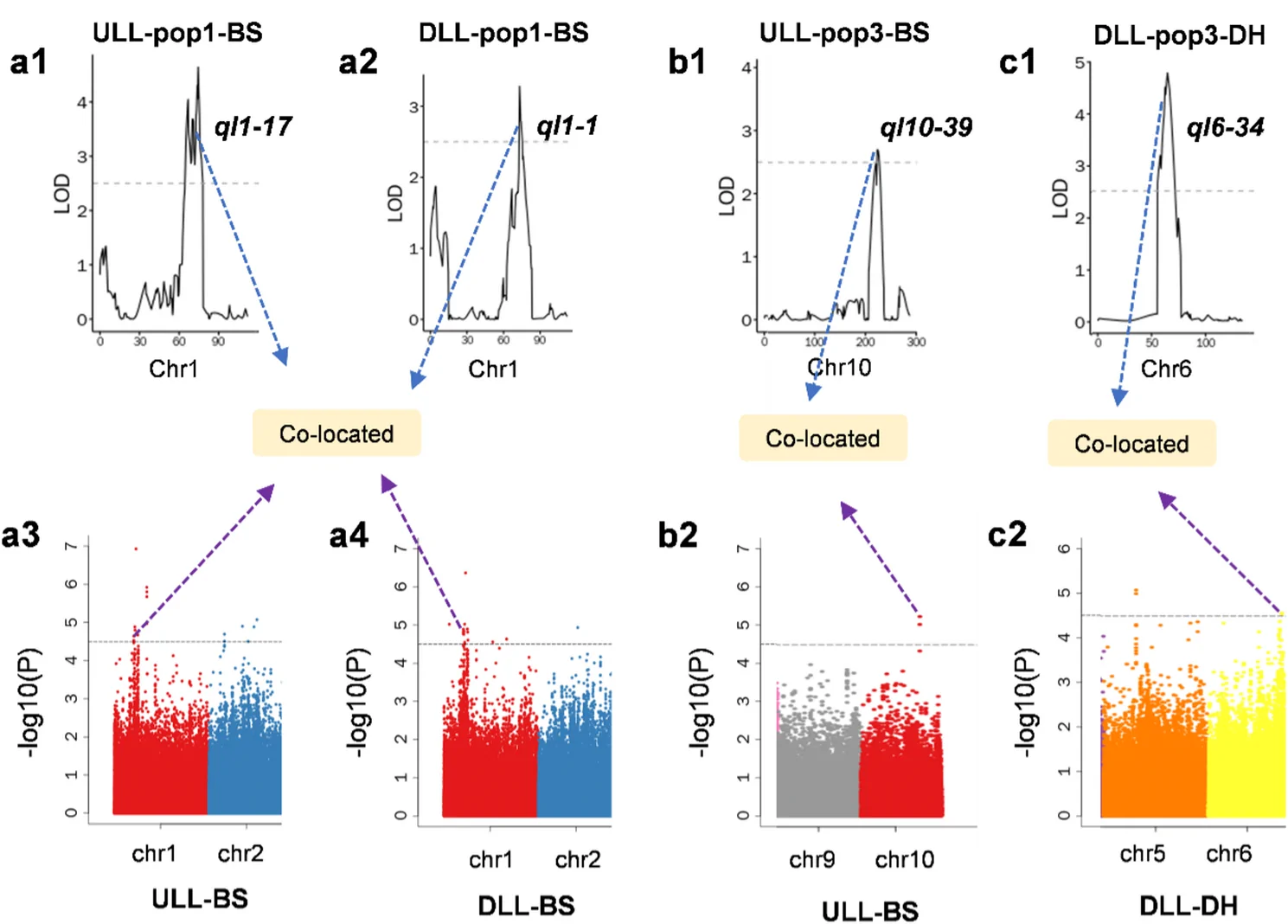
Colocalization of significant SNPs identified by GWAS and QTL intervals. (a1-a5) Colocalization of SNP1- 63031255 and SNP1-63031328 within the QTL intervals *ql1-17* and *ql1-1*; (b1-b2) colocalization of SNP10-110436440 within the QTL interval *ql10-39*; (c1-c2) colocalization of SNP 6-155639903 within the QTL interval *ql6-34*

A total of 7 QTL were detected for upper leaf length in Pop1 and Pop3(Fig. S1a). In Pop1, for the 19BS environment, a QTL cluster was identified on chromosome 1, consisting of five closely linked loci (*ql1-17*,* ql1-13*,* ql1-12*,* ql1-9*,* ql1-8*). These loci were detected within an interval of 69.4-72.84 cM (approximately 62–81 Mb), and each explained more than 9% of the phenotypic variation, indicating that they can be considered major QTL. Among them, *ql1-17* showed the largest effect, explaining 12.10% of variation. In addition, a significant QTL, *ql1-1*, was detected at 4.2 cM on chromosome 1 based on BLUP values. For ear leaf length in Pop3, a dominant QTL *ql6-15* was identified in the 19BS environment on chromosome 6 at 121.69 cM (approximately 36 Mb-41 Mb)༈Fig. S1e༉. This QTL showed a high LOD value of 4.57 and explained 12.82% of the phenotypic variation. For lower leaf length, four QTL were detected on chromosomes 1 and 6. In the Pop1, BLUP data analysis mapped *ql6-12* on chromosome 6, explaining 8.74% of the phenotypic variation༈Fig. S1g༉. In the Pop3, the QTL *ql6-34* on chromosome 6 exhibited a strong genetic effect, with a LOD value of 4.55, and 11.84% phenotypic variation explained in the 18DH environment (Fig. S1h).

Colocalization analysis of QTL across traits revealed several co-localized regions. In Pop1, the region near 72.84 cM on chromosome 1 was associated with both ULL (*ql1-17*) and DLL (*ql1-1*). Similarly, in the BLUP analysis of Pop1, overlapping QTL affecting ULL (*ql1-1*) and DLL (*ql1-2*) were detected at 4.2 cM on chromosome 1. These colocalizations suggest the presence of pleiotropic loci or tightly linked gene controlling leaf length at multiple plant positions.

The environmental stability of QTL was evaluated based on LOD (A×E), PVE (A×E), and the ratio PVE(A×E)/PVE(A) (Table S5). None of the detected QTL exhibited significant QTL × environment interactions, as all LOD (A×E) values were below the significance threshold. QTL such as *ql6-15*, *ql10-39*, *ql1-9*, and *ql6-12* showed low interaction contributions (< 10%), indicating high environmental stability. QTL including *ql1-17*, *ql1-13*, *ql1-1* (Chr1:4 cM), and *ql1-2* exhibited moderate interaction effects (e.g., 14.2% for *ql1-17*), indicating predominantly additive genetic control with minor environmental influence. In contrast, *ql8-3* and *ql1 -1* (Chr 1:73 cM) showed relatively high (A×E)/PVE(A) (46% and 54%), indicating greater environmental sensitivity, although the interaction effects were not statistically significant. Overall, leaf length in this population was primarily controlled by additive genetic effects, with limited QTL × environment interaction. Highly stable QTL, particularly, *ql6-15*, *ql10-39*, and *ql1-17* (level I and II), represent promising targets for breeding programs requiring broad environmental adaptability, whereas environmentally sensitive loci (level III) may be useful for region-specific genetic improvement under specific ecological conditions.

**Table 4:** List of QTL associated with leaf length (LL) in maize

Trait	Population	Environment	Chr	QTL	Position (cM)	Physical interval (bp)	LOD	*R*^2^ (%)
ULL	Pop1	19BS	1	*ql1-17*	72.84	62,780,279–63,359,030	3.75	12.10
			1	*ql1-13*	70.96	65,636,228–66,433,802	3.63	11.76
			1	*ql1-12*	70.65	66,433,803–67,364,677	3.68	11.90
			1	*ql1-9*	69.71	69,802,996–71,520,084	3.44	11.18
			1	*ql1-8*	69.4	71,520,085–81,723,312	2.93	9.68
		18DH	8	*ql8-3*	0.01	175,433,797–181,122,637	3.09	1.03
		BLUP	1	*ql1-1*	4.2	275,031,928–284,429,734	2.51	5.86
	Pop3	19BS	10	*ql10-39*	222.83	110,324,107–111,215,014	2.57	6.74
ELL	Pop3	19BS	6	*ql6-15*	121.69	36,223,458–41,341,179	4.57	12.82
DLL	Pop1	19BS	1	*ql1-1*	72.84	62,780,279–63,359,030	2.66	8.63
		BLUP	1	*ql1-2*	4.2	275,031,928–284,429,734	2.64	6.12
			6	*ql6-12*	31.23	103,976,806–105,369,354	3.65	8.74
	Pop3	18DH	6	*ql6-34*	66.1	154,239,769–156,482,302	4.55	11.84

### Candidate gene identification based on GWAS and QTL mapping co-localization and Functional Annotation

**Fig. 9:**
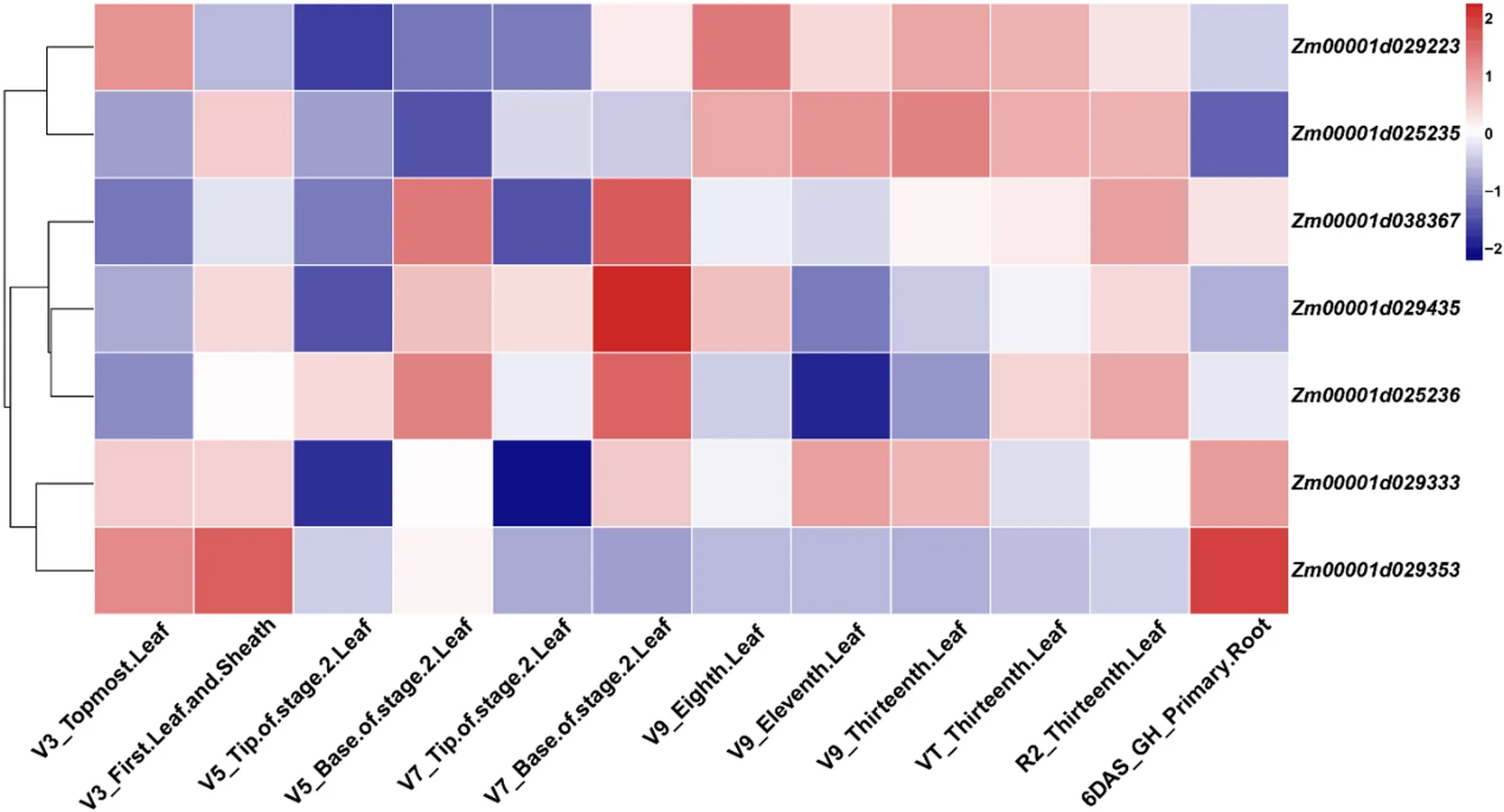
The heat map showing the expression profiles of candidate genes based on the B73 RefGen_v4 expression database. The x-axis represents different maize developmental stages, leaf positions, and tissues, with taproot tissue collected at 6 days after sowing (6 DAS) used as the reference. The y-axis represents the candidate genes. The color scale indicates relative expression levels, where red indicates upregulation, and blue indicates downregulation

By integrating significant SNPs identified by GWAS with QTL intervals, genomic regions showing physical colocalization were defined as primary targets for candidate gene identification for maize leaf length traits (Fig. 9; Table 5).

Colocalization analysis showed significant overlapping on chromosomes 1, 6 and 10, which were detected by both GWAS and QTL mapping, suggesting stable genetic effects in regulating leaf development. On chromosome 1, two significant SNPs (1-63031255 and 1-63031328) were located within the overlapping intervals of QTL *ql1-17* (ULL, Pop1, 19BS environment) and *ql1-1* (DLL-Pop1-19BS environment) (Fig. 8a1-a4). This cross-trait colocalization suggests that this region may have potential pleiotropic or tight linkage effects on leaf elongation at different nodes. On chromosome 10, SNP 10-110436440, significantly associated with upper leaf length, was located within the physical interval of QTL *ql10-39* (ULL, Pop3, 19BS environment) (Fig. 8b1-b2). In addition, for lower leaf length, SNP 6-155639903 located on chromosome 6 was colocalized with the major QTL *ql6-34* (DLL, Pop3, 18DH environment) (Fig. 8c1-c2).

Based on these colocalized regions and gene annotation using the maize B73 RefGen_v4 genome, three candidate genes were identified (Table 5). To further evaluate their potential biological roles, expression profiles of *ZmHUA2*, *ZmCAMTA5* and *ZmPAT16* were analyzed using public transcriptome data (B73 RefGen_v4 expression database) (Fig. 9). All three genes showed spatiotemporal expression patterns associated with the vegetative-to-reproductive transition and leaf elongation stages. *ZmHUA2* exhibited relatively high expression in the upper leaves during the V9–VT stages but low expression in early seedling leaves and roots. *ZmCAMTA5* showed high expression in middle and late leaves with variation among leaf positions. *ZmPAT16* showed upregulation in leaves at specific developmental stages during key developmental periods. These expression patterns were consistent with the critical stages of leaf growth and support their potential roles in leaf length elongation. Functionally, *ZmHUA2* located within the colocalized interval of chromosome 1, encodes ENHANCER OF AG-4 protein 2 (HUA2) and was associated with both upper and lower leaf length. HUA2 has been reported to participate in floral development and transcriptional regulatory networks involved in developmental phase transition [70]. It is speculated that this gene may play a role in coordinating the transition stage from vegetative-to-reproductive transition and leaf morphology. The candidate gene *ZmCAMTA5*, identified on chromosome 10, encodes a calmodulin-binding transcription activator 5, indicating a potential role in regulating upper leaf development through calcium signaling pathway [71, 72]. *ZmPAT16*, located within the interval of *ql6-34* chromosome 6, encodes protein S-acyltransferase 16. Protein S-acylation is an important post-translational modification involved in membrane localization and signal transduction during plant growth and development [73], suggesting a role in the regulation of lower leaf length.

Overall, the integration of GWAS and QTL mapping improved the reliability of detected loci and provided high-confidence candidate genes for elucidating the molecular mechanisms underlying leaf length variation at different nodes of maize.

**Table 5:** Candidate genes identified through colocalization by QTL mapping and GWAS

Trait	Chr	SNP	QTL	Candidate gene	Candidate gene Range (bp)	Encoded protein
ULL	1	1- 63,031,255	*ql1-17*	*ZmHUA2*	63,025,344–63,042,076	ENHANCER OF AG-4 protein 2
	10	10- 110,436,440	*ql10-39*	*ZmCAMTA5*	110,432,656–110,441,915	Calmodulin-binding transcription activator 5
DLL	1	1- 63,031,328	*ql1-1*	*ZmHUA2*	63,025,344–63,042,076	ENHANCER OF AG-4 protein 2
	6	6-155639903	*ql6-34*	*ZmPAT16*	155,628,842–155,636,644	Protein S-acyltransferase 16

### Haplotype analysis

**Fig. 10:**
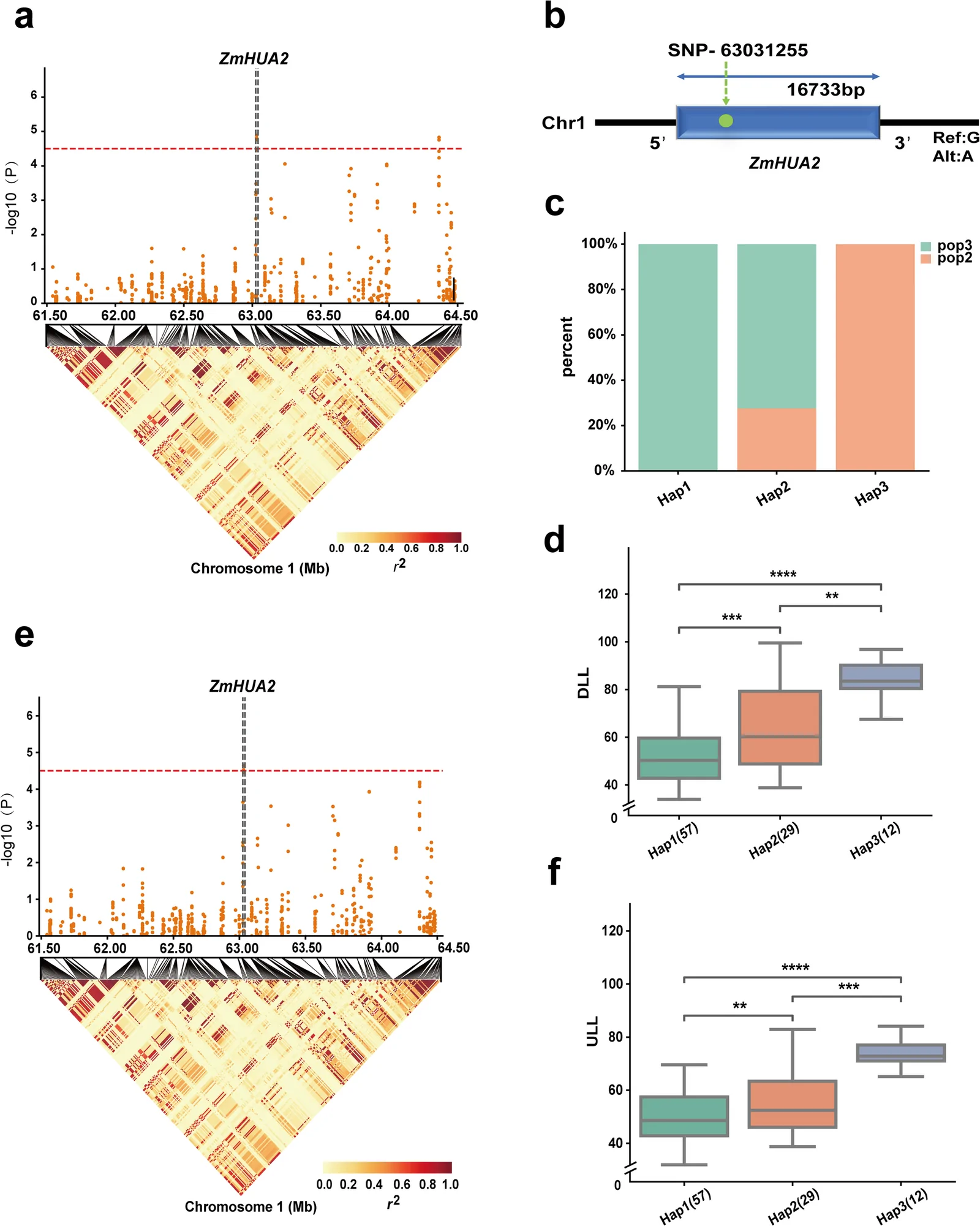
Haplotype analysis of the candidate gene *ZmHUA2*. (**a**) LD block in the genomic region associated with lower leaf length; (**b**) genomic position of SNP1- 63,031,255 within the candidate gene; (**c**) frequency distribution of the three haplotypes in the MPP; (**d**) phenotypic differences among haplotypes for lower leaf length; (**e**) LD block in the genomic region associated with upper leaf length; (**f**) phenotypic differences among haplotypes for upper leaf length. Statistical significance among haplotypes was determined using one-way ANOVA followed by multiple comparison tests. The number of lines in each haplotype group is indicated in the figure. ** indicate significant differences (**P < 0.01; ***P < 0.001; ****P < 0.0001)

To further evaluate the genetic effect of the key candidate gene *ZmHUA2* on leaf length traits, haplotype analysis was performed based on the significant associated SNP, SNP1-63031255 (Fig. 10). LD analysis revealed strong LD in the genomic region flanking this SNP (Fig. 10a, e).

Based on the SNP variation within this genomic region, three major haplotypes were identified in the MPP, designated Hap1, Hap2, and Hap3 (Table 6; Fig. 10c). The haplotype frequencies were 58.16% for Hap1, 29.6% for Hap2, and 12.24% for Hap3. Association analysis using phenotypic data showed significant differences among haplotypes. For lower leaf length, plants carrying the Hap3 haplotype exhibited the longest leaves, with mean values significantly higher than those of Hap1 and Hap2, whereas Hap1 showed the shortest length (Fig. 10d). A similar pattern was observed for upper leaf length, where Hap3 was associated with significantly greater leaf length compared with the other haplotypes (Fig. 10f). These results indicate that Hap3 represents a favorable haplotype with positive effects on leaf elongation. Since *ZmHUA2* encodes ENHANCER OF AG-4 protein 2 (HUA2), the observed haplotype variation may regulate leaf development by affecting regulatory processes related to cell proliferation or elongation.

For the candidate gene *ZmCAMTA5*, two major haplotypes (Hap1 and Hap2) were identified based on SNP variation in the coding and promoter regions. Although the mean leaf length of Hap1 was slightly higher than that of Hap2, the difference was not statistically significant (Fig. S2). No polymorphic variants were detected for *ZmPAT16* in the MPP. Haplotype analysis indicated that this gene is highly conserved, suggesting that natural sequence variation at this locus is unlikely to be a major contributor to leaf length variation.

**Table 6:** Haplotype analysis of *ZmHUA2* in relation to leaf length in maize

SNP	Gene ID	Gene-Start	Gene-End	Haplotype	Frequency (%)
1- 63,031,255	*ZmHUA2*	63,025,344	63,042,076	GAGCG(Hap1)	58.16
				GTATA(Hap2)	29.60
				ATACA(Hap3)	12.24

### Analysis of the relative expression of candidate genes

**Fig. 11:**
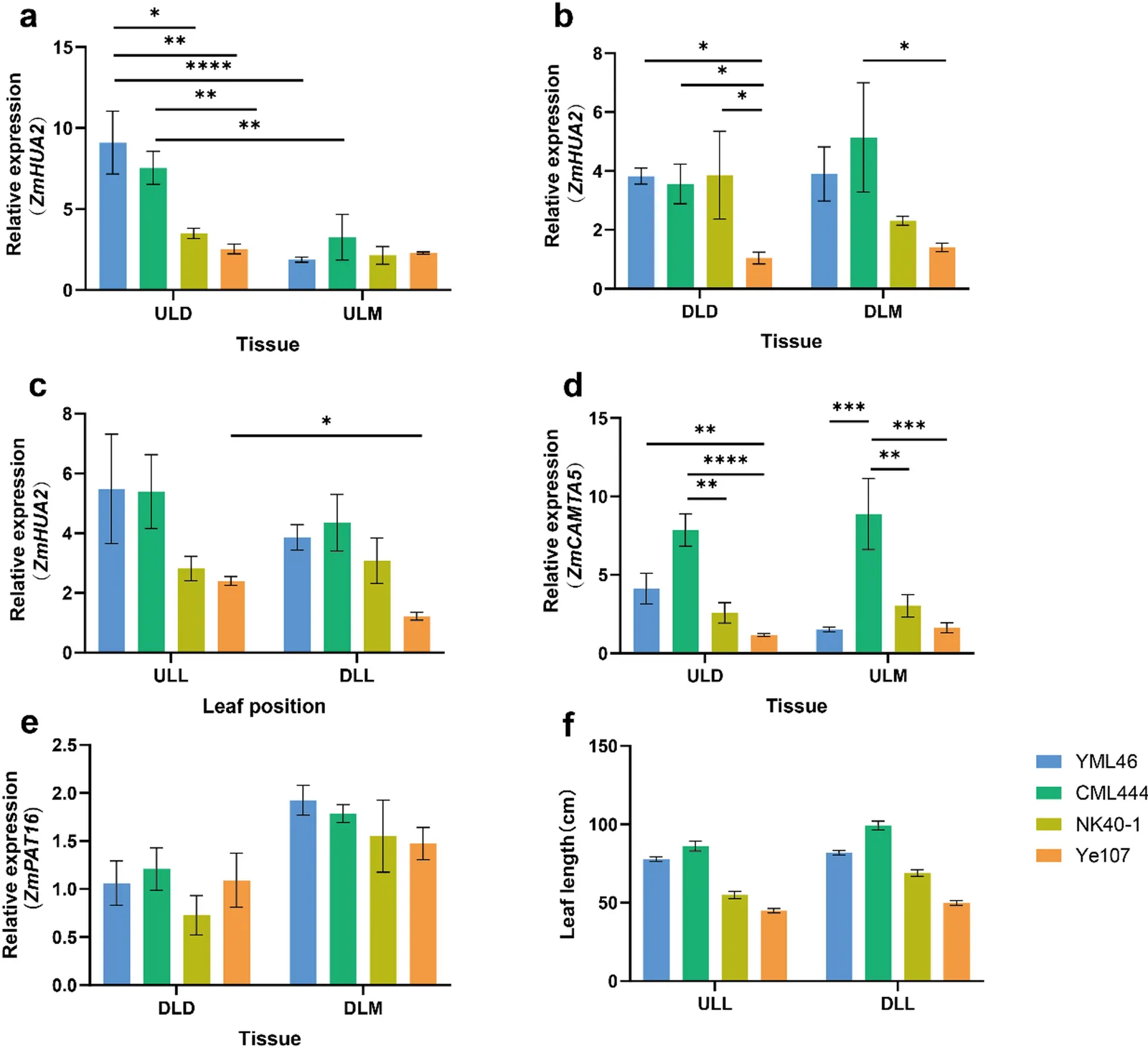
Relative expression analysis of three candidate genes at different leaf positions and developmental regions in the four parental lines. (**a**) Expression of *ZmHUA2* in the upper leaf base (ULD) and middle segment (ULM); (**b**) expression of *ZmHUA2* in the basal and middle regions of lower leaves; (**c**) overall expression of *ZmHUA2* in upper and lower leaves; (**d**) expression of *ZmCAMTA5* in the basal and middle regions of upper leaves; (**e**) expression levels of *ZmPAT16* in the basal and middle regions of lower leaves; (**f**) leaf length phenotypes in the upper and lower leaves of different parents. Data are presented as mean ± SD from three biological replicates. Statistical significance was determined using Student’s t-test (*P < 0.05, **P < 0.01, ***P < 0.001, ****P < 0.0001)

The candidate gene *ZmHUA2* showed significant differences in spatial and developmental stage-specific expression among the four parental lines (Fig. 11a-c). In upper leaves, the expression of this gene in the middle segment (ULM) of the long-leaf parents was significantly higher than that in the basal segment (ULD) and was also significantly higher than that of the short-leaf parent Ye107 (Fig. 11a), suggesting a potential role in the cell elongation process of the upper leaf. A similar pattern was observed in lower leaves, where differences in expression among parents were most significant in the elongation region (Fig. 11b). Furthermore, comparison of overall expression levels across different leaf positions (Fig. 11c) indicated that gene expression at specific developmental stages was positively associated with the length phenotype of the parental lines (Fig. 11f), supporting its potential role in regulating maize leaf elongation. The candidate gene *ZmCAMTA5* exhibited a specific expression pattern related to upper leaf development. As shown in Fig. 11d, significant differences in expression were observed among parental lines, especially in the elongation region that determines final leaf length. The expression level in the long-leaf parent was significantly higher than that in Ye107, indicating that *ZmCAMTA5* may be involved in upper leaf elongation. In contrast, the candidate gene *ZmPAT16* showed no significant differences in expression among parental lines or between tissue regions (Fig. 11e). Although this gene was expressed in leaf tissues, its expression levels were similar between long- and short- leaf parents, as well as between basal and middle segments.

Overall, *ZmHUA2* and *ZmCAMTA5* exhibited diverse spatiotemporal expression patterns during maize leaf development that were consistent with phenotypic differences among parental lines. The high expression of these genes in the elongation regions of long-leaf parents suggests their potential involvement in the positive regulation of leaf length.

## Discussion

### Comparative analysis of leaf length-related loci with previous studies (upper leaf length)

The upper leaf length plays an important role in canopy structure by affecting light penetration within the upper canopy layer. Excessively long upper leaves may shade the middle and lower leaves, thereby reducing the photosynthetic efficiency of the population. In this study, multiple key loci associated with ULL were identified on chromosomes 1, 8, and 10. On chromosome 1, a major QTL cluster (*ql1-17*) was detected within the 62.78–63.35 Mb interval (69.4–72.84 cM), explaining up to 12.10% of the phenotypic variation in Pop1 in the 19BS environment. Previous studies have reported QTL on chromosome 1 associated with leaf length and plant architecture traits using different populations and analytical methods. For example, Pan et al. (2017) identified several genomic regions contributing to leaf length in multiple RIL populations [74]. A recent GWAS by Zeng et al. (2025) also detected significant association signals on this chromosome [28]. However, the intervals reported in these studies were relatively broad and were not precisely localized to *ql1-17* region identified here, suggesting that *ql1-17* may represent a novel and stable regulatory region that warrants further fine mapping and functional validation. The discrepancy in the phenotypic variance explained (PVE) between the GWAS tag SNP (1-63031255, PVE = 0.1%) and the colocalized major QTL (*ql1-17*, PVE = 12.10%) highlights the complex genetic architecture of leaf length. While the MLM in GWAS rigorously controls for false positives, it often underestimates the effect size of single markers. Conversely, the QTL interval encompasses a much broader linkage block, suggesting that *ql1-17* may be driven by a cluster of tightly linked loci contributing cumulatively to the 12.10% phenotypic variation. In addition, *ql1-1* was detected at 4.2 cM within the 275.03–284.42 Mb interval of chromosome 1 based on BLUP values. This region overlapped with the significant SNP1-279508495 identified by GWAS. Previous studies have also reported important plant architecture loci in this region. For example, Yang et al. (2014) identified a major locus controlling plant height at 285.93 Mb [75], and Tian et al. (2011) reported a key QTL for leaf length on the long arm of chromosome 1 [33]. The consistency of these findings supports the reliability of *ql1-1* and suggests that this region may affect leaf length though its effects on plant elongation.

On chromosome 10, localizes *ql10-39* (110.32–111.21 Mb; 222.83 cM) was identified in the Pop3 subpopulation, explaining 6.74% of the phenotypic variation. This QTL is located near previously reported leaf architecture QTL clusters (e.g., *qLW-10-2* and *qLAr-10-2*) identified in the IBM population by Ma et al. (2018), within the genetic hotspot region of bin 10.04. This region contains the well-known flowering time regulatory gene *ZmCCT*, and a variation in this region may indirectly affect upper leaf length by influencing overall plant developmental timing [75]. The *ql8-3* locus on chromosome 8 (175.43–181.12 Mb, 0.01 cM) explained a relatively small proportion of phenotypic variation (1.03%) but showed strong stability across environments. This result is consistent with previous findings by Yang et al. (2016), who reported that the distal region of the long arm of chromosome 8 (bin 8.05) contains conserved genetic elements regulating leaf development and exhibits stable environmental effects [76] .

### Comparative analysis of genetic loci for lower leaf length with previous studies

The lower leaves are more prone to shading during the middle and late growth stages, which can trigger shade-avoidance responses and leads to excessive leaf elongation [77]. The genetic analysis revealed that lower leaf length shares common regulatory regions with upper leaf length. Overlapping QTL intervals were detected on chromosome 1 within the 62.78–63.35 Mb (*ql1-1*) and 275.03–284.42 Mb (*ql1-2*) intervals, indicating potential pleiotropic effects or tight linkage between loci controlling leaf elongation at different plant positions. A similar pattern was reported in previous studies. For example, Tian et al. (2011) identified 36 leaf length QTL using a NAM population, several of which were located near bin 1.05 and showed coordinated effects on leaf architecture [33]. These findings suggest that key regions on chromosome 1 may play a central role in regulating longitudinal leaf development across the whole plant.

The lower leaf length also showed position-specific genetic control. On chromosome 6, a major QTL (*ql6-34*) was detected within the 154.23–156.48 Mb interval, explaining 11.84% of the phenotypic variation. This interval overlaps with *qMLN06-157*, a major locus associated with the maize lethal necrosis (MLN) resistance, and is located near genes related to leaf senescence and stress responses reported by Murithi et al. (2021) [78]. The colocalization suggests that natural variation in lower leaf length may be associated with stress adaption processes, potentially through effects on cell wall properties, membrane stability, or signal transduction. In addition, a significant SNP (SNP3-206954273) on chromosome 3 showed a strong positive additive effect (3.10). This region corresponds to a previously reported hotspot near bin 3.05 associated with internode elongation [79], indicating that common growth-regulatory mechanisms may influence both stem and leaf elongation.

While our study identified several stable QTL across two environments, it is important to acknowledge that two environments may not fully capture the complete spectrum of G×E interactions. However, the high heritability and strong cross-environmental correlation observed for leaf length traits suggest that the identified major loci are reliable. Further validation across a wider range of ecological zones will be essential to confirm the broad adaptability of these superior haplotypes for molecular design breeding.

#### Comparative analysis of candidate genes

*ZmHUA2* was annotated as ENHANCER OF AG-4 protein 2 (HUA2). In *Arabidopsis thaliana*, HUA2 is a multifunctional regulator involved in floral organ development and the transition from vegetative to reproductive growth through regulation of pre-mRNA splicing and transcriptional processing [80, 81]. In the present study, this gene was associated with both upper and lower leaf length, suggesting a broader role in maize vegetative organ development. Previous studies of maize leaf development have mostly focused on the classical regulators such as liguleless (*lg1*, *lg2*) and Growth-Regulating Factor (GRF) genes [82, 83]. The identification of HUA2 expands the regulatory framework of leaf length from hormone signaling and developmental regulators to include RNA processing and developmental phase transition mechanisms. Unlike *lg1* which primarily affects leaf angle, HUA2 in this study showed a strong association with longitudinal leaf elongation.

The candidate gene *ZmCAMTA5* on chromosome 10 encodes calmodulin-binding transcriptional activator 5 (CAMTA5). Calcium signaling pathways play a central role in plant stress responses and organ growth. Members of the CAMTA family regulate the expression of downstream growth-related genes in responses to changes in intracellular Ca^+ 2^ levels [84]. In this study, CAMTA5 was specifically associated with upper leaf length, suggesting that calcium-mediated transcriptional regulation contributes to leaf elongation. Compared with previously reported leaf development regulators such as *ZmEREBP60* and auxin-responsive SAUR genes [69, 85], CAMTA5 may function as an environmentally responsive regulator. This mechanism may partly explain the relatively stable expression and phenotypic performance of upper leaf length across environments compared with lower leaf length.

#### Candidate gene functional analysis

The gene *ZmHUA2*, encoding HUA2, contains a PWWP domain and has been reported to regulate the transition from vegetative to reproductive growth in *Arabidopsis thaliana* through the modulation of pre-mRNA splicing of key developmental genes such as *FLC* and *AG* [80, 86]. In this study, expression analysis (Fig. 9) showed that, *HUA2* was highly expressed in the V9–VT stages, a critical period for the vegetative-to-reproductive transition. This upregulation was particularly prominent in ear and upper leaves. From a cytological perspectives, maize leaf length is largely determined by the activity of the basal cell division zone (DZ) [87]. The superior haplotype Hap3, which is associated with increased leaf length, may enhance leaf elongation by increasing the proliferative capacity of the division zone. This effect could be achieved through upregulation of *HUA2*, leading to prolonged cell residence time in the division region or the activation of cell cycle-related genes such as *Cyclins*. This dual mechanism, involving both developmental phase regulation and accelerated cell division, may explain the effects of this gene on both upper and lower leaf length.

The candidate gene *ZmCAMTA5*, encoding calmodulin-binding transcription activator 5 (CAMTA5), showed specific association with upper leaf length and higher expression in the elongation region of long-leaf parents. Upper leaves endure intense illumination and higher transpirational pull, presenting a stark contrast to the shaded conditions of the lower canopy [88]. As calmodulin-binding transcription factor, members of the CAMTA family have been reported to participate in calcium-mediated signal transduction and environmental stress responses in plants [84]. We hypothesize that CAMTA5 may participate in the regulation of upper leaf elongation by mediating calcium signaling in response to fluctuating light or water status, thereby regulating cell wall extensibility or auxin sensitivity. This mechanism may contribute to canopy-level plasticity, allowing maize plants to optimize light interception under variable environmental conditions. However, this inference is currently based on the expression patterns and environmental associations, and requires further experimental validation.

Although expression analyses were conducted at comparable developmental stages, gene expression patterns may still be influenced by developmental dynamics and environmental conditions. Therefore, broader spatiotemporal analyses across multiple growth stages and environments will be necessary to further validate the regulatory roles of these candidate genes.

While the identification of these candidates provides an important foundation, establishing causality will require functional validation. Future studies should therefore prioritize the genetic characterization of *HUA2* and *CAMTA5* through CRISPR/Cas9-based knockout and overexpression approaches. Detailed cytological analyses within the elongation zone may help clarify how these genes influence cell expansion dynamics. In parallel, combining phenotypic observations with yeast two-hybrid (Y2H) assays and chromatin immunoprecipitation sequencing (ChIP-seq) analyses could provide deeper insight into the regulatory networks underlying maize leaf development.

#### Analysis of genotype stability and application in temperate breeding

In this study, Hap3 was confined to the Pop2 subpopulation and traced back to the tropical parent CML444. Its absence from the other subpopulations may reflect historical differences in breeding history between tropical and temperate germplasm pools. Such contrasts in allele distribution further underscore the value of tropical genetic resources for broadening the allelic spectrum available in temperate maize improvement.

To evaluate the practical application value of Hap3 in breeding, we analyzed its introgression potential. The significant phenotypic effect exhibited by this haplotype, particularly its coordinated promotion of both upper and lower leaf elongation, makes it a powerful genetic tool for “remodeling” the canopy structure of traditional temperate inbred lines. In future molecular design breeding, breeders can accurately track the introgression of Hap3 into different temperate genetic backgrounds (such as the Reid or Non-Reid groups) by utilizing the diagnostic marker SNP1-63031255.

This strategy will enable breeders to effectively compensate for the “genetic degradation” of leaf size caused by extreme density-tolerance screening. By introducing this increasing allele, it is possible to achieve an optimal balance between individual plant photosynthetic capacity and population density tolerance by optimizing leaf length at specific nodes while maintaining a compact plant type, thereby tapping into higher yield potential.

### Breeding value of Ye107 in relation to leaf length phenotype

We utilized Ye107, a widely planted compact inbred line central to Chinese breeding and hybrid density tolerance, as our common parent. While tropical donor lines frequently produce upper leaves well over 80 cm, upper leaf length in Ye107 averages around 45 cm. This stark difference in leaf architecture made it an excellent temperate foundation for our crosses. By introducing long-leaf tropical traits into this compact background, we effectively expanded the genetic diversity of temperate materials and captured novel alleles driving extreme variations in leaf elongation.

Temperate Reid germplasm has undergone intensive artificial selection, which has likely reduced allelic diversity for traits related to ideal plant architecture (IPA) [26, 89]. By hybridizing Ye107 with diverse tropical lines, favorable alleles for growth and leaf development that are absent or rare in Ye107 were captured in the derived population. Notably, some tropical donors, despite their long-leaf phenotype, contributed haplotypes that improved plant compactness at specific loci. Such recombination generates transgressive segregants with superior phenotypes beyond those of the parents.

Previous studies by our research group demonstrated that Ye107 can serve as a backbone parent for hybrid development. Several improved varieties, such as Yunrui 88 and Yunrui 7, were successfully developed through the introgression of superior tropical haplotypes. The favorable HUA2 Hap3 identified in this study may represent one of the genetic factors contributing to improved canopy performance under high-density planting conditions. These results indicate that precise genetic dissection of leaf length traits can facilitate the development of functional markers for marker-assisted selection (MAS), and provide tools for optimizing maize canopy architecture.

However, our results indicate that Hap3 exerts a coordinated promotive effect on the elongation of both upper leaves (ULL) and lower leaves (DLL). Direct introgression of this haplotype into temperate density-tolerant lines such as Ye107 may improve light interception in the lower canopy, but it could also result in excessive elongation of upper leaves, thereby aggravating canopy shading at the population level. Therefore, the direct utilization of this haplotype in future molecular design breeding may face the challenge of simultaneous increases in leaf length across different nodal positions. Future breakthroughs may depend on either disrupting the tight linkage within this genomic region through larger mapping populations or identifying downstream target genes that specifically regulate lower leaf length (DLL). Such strategies would enable the genetic decoupling of leaf length among different nodes, thereby providing precise molecular tools for reshaping a high-efficiency canopy architecture with improved light utilization.

## Conclusion

In this study, a multi-parent population derived from temperate and tropical germplasm was used to dissect the genetic basis of maize leaf length. By integrating GWAS and QTL mapping, several key loci regulating leaf length were identified, and the genetic architecture of leaf development at different plant nodes was systematically analyzed. Our findings suggest that maize leaf length is controlled by both shared and node-specific genetic effects. Several major genomic regions, particularly on chromosome 1, were repeatedly detected across leaf positions, whereas additional QTL appeared to act in a node-dependent manner. Among the candidate genes identified, *ZmHUA2 (Zm00001d029223)* and *ZmCAMTA5 (Zm00001d025235)* are likely associated with developmental phase transitions and signaling processes. Notably, favorable haplotypes derived from tropical parents contributed positively to leaf elongation, underscoring the value of tropical germplasm for enriching the temperate genetic background and optimizing plant architecture. Overall, the major QTL and favorable alleles identified in this study provide valuable genetic resources for molecular breeding and the optimization of maize canopy structure, facilitating the development of high-density, high-yield maize ideotypes.

## Supplementary Information

Supplementary Material 1.

Supplementary Material 2.

Supplementary Material 3.

Supplementary Material 4.

Supplementary Material 5.

Supplementary Material 6.

Supplementary Material 7.

## Data Availability

The raw sequencing data generated in this study have been deposited in the Genome Sequence Archive (GSA) at the National Genomics Data Center under accession number CRA026598 ( https://ngdc.cncb.ac.cn/gsa/browse/CRA026598 ).

## Abbreviations

ULL: Upper Leaf Length
ELL: Ear Leaf Length
DLL: Lower Leaf Length
LL: Leaf Length
IPA: Ideal Plant Architecture
SNP: Single Nucleotide Polymorphism
QTL: Quantitative Trait Loci
GWAS: Genome-wide Association Study
GBS: Genotyping-by-Sequencing
BLUP: Best Linear Unbiased Prediction
LD: Linkage Disequilibrium
CDS: Coding Sequence
qRT: PCR-Quantitative Real-time PCR
