# Supplementary material for: Genetic architecture of node-specific leaf length and candidate gene identification in maize revealed by a temperate–tropical multiparental population
Source: BMC Plant Biol. 2026 Aug 4;26:1864. doi: 10.1186/s12870-026-09606-3 (PMC13726121; doi:10.1186/s12870-026-09606-3)
Supplement: Supplementary file 1 [file 12870_2026_9606_MOESM4_ESM.pdf]

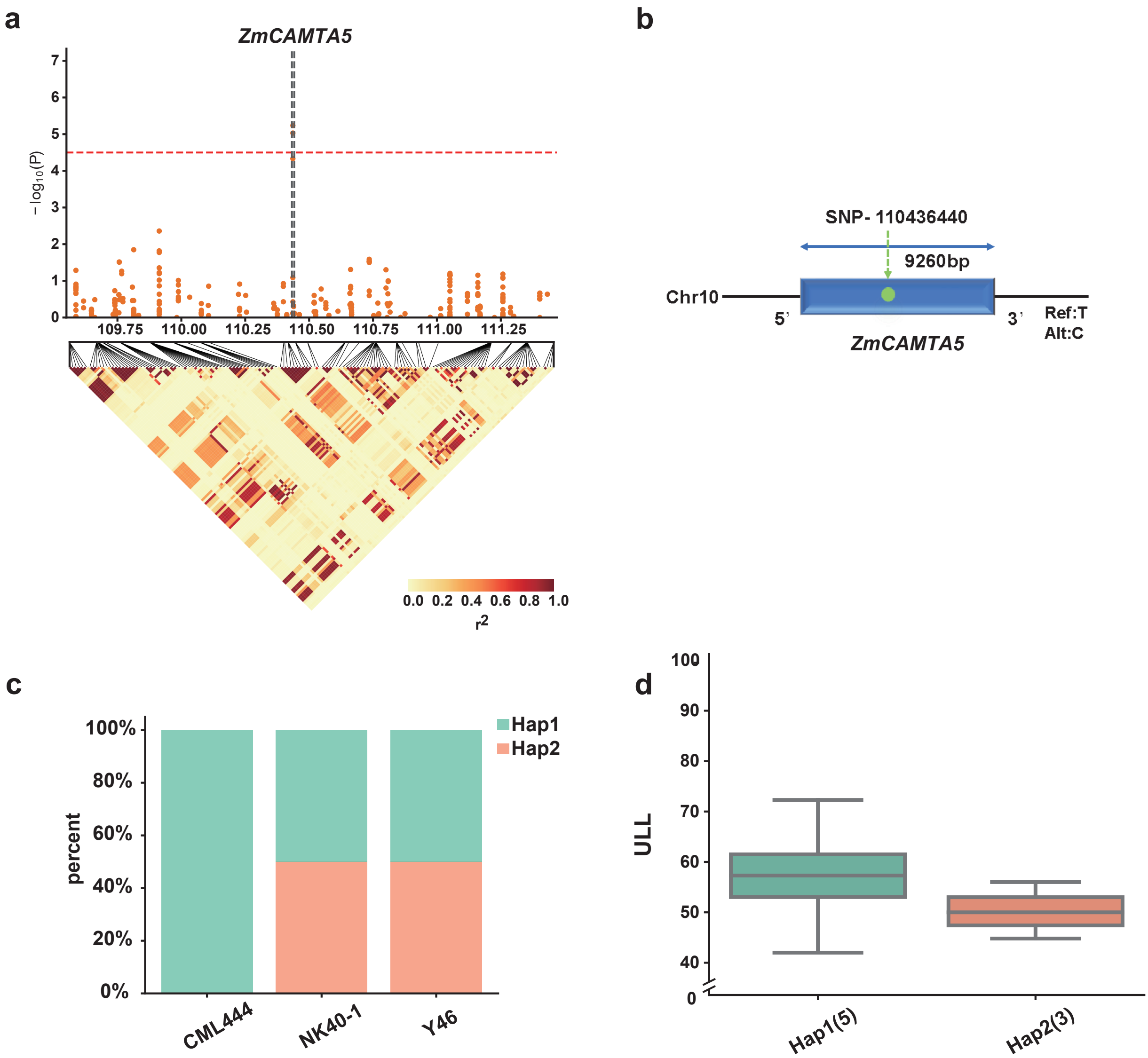

Fig. S1 Haplotype analysis of the candidate gene *ZmCAMTA5*. **a** LD block in the genomic region associated with lower leaf length; **b** genomic position of SNP10- 110436440 position within the candidate gene; **c** Frequency distribution of the two haplotypes in the MPP; **d** phenotypic differences among haplotypes for lower leaf length.
