# Supplementary material for: Genetic architecture of node-specific leaf length and candidate gene identification in maize revealed by a temperate–tropical multiparental population
Source: BMC Plant Biol. 2026 Aug 4;26:1864. doi: 10.1186/s12870-026-09606-3 (PMC13726121; doi:10.1186/s12870-026-09606-3)
Supplement: Supplementary file 2 [file 12870_2026_9606_MOESM7_ESM.pdf]

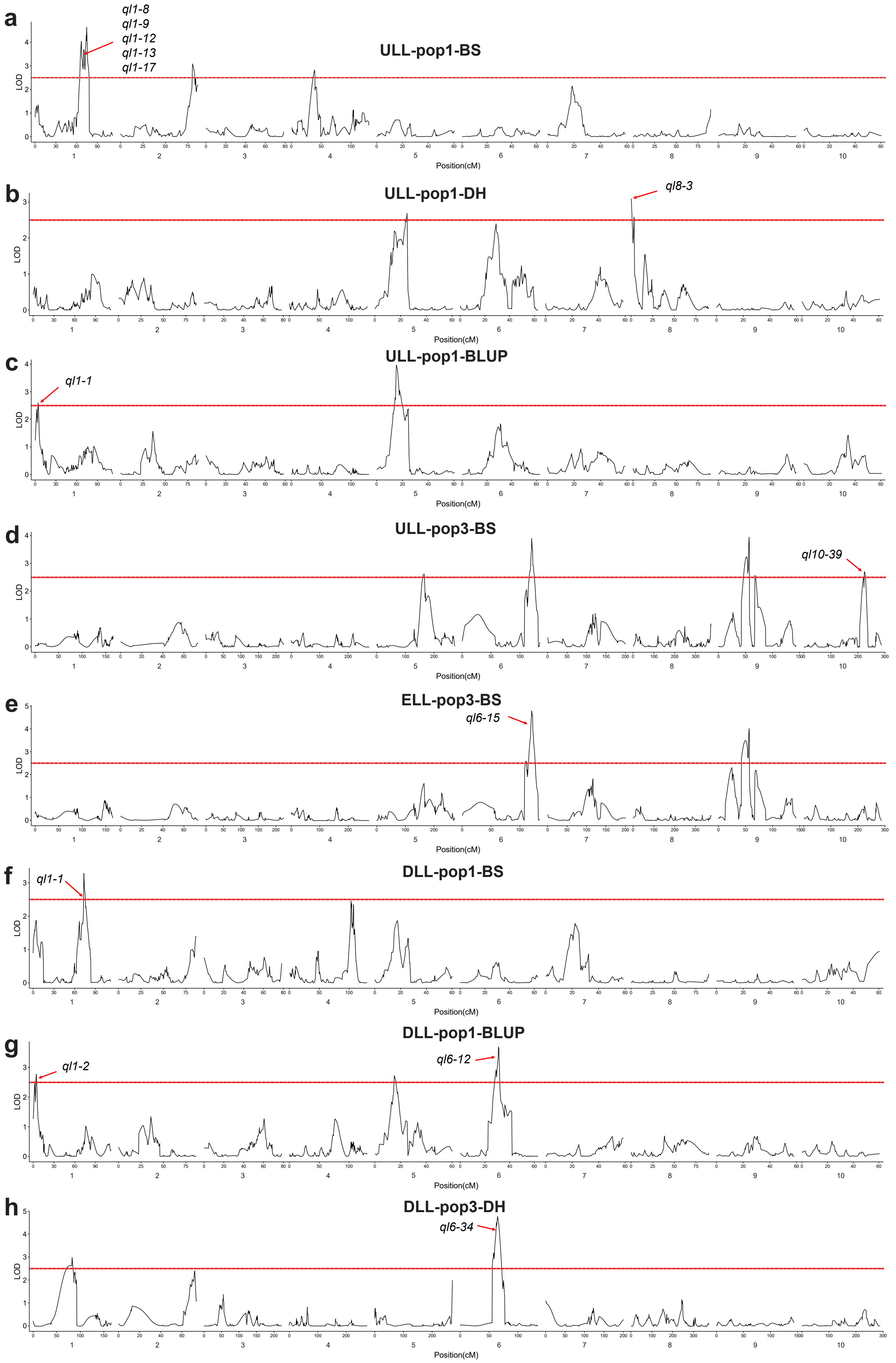

Fig. S1 QTL mapping of leaf length traits in different populations. (a-c) QTL for upper leaf length (ULL) in Pop1 in the 19BS environment (a), 18DH environment (b), and BLUP analysis (c); (d) QTL for upper leaf length (ULL) in Pop3 in the 19BS environment; (e) QTL for ear leaf length (ELL) in Pop3 in the 19BS environment; (f-g) QTL for lower leaf length in Pop1 in the 19BS environment (f), and BLUP analysis (g); (h) QTL for lower leaf length (DLL) in Pop3 in the 18DH environment.
